# Inhibition of Influenza Virus Polymerase by Interfering with Its Protein–Protein Interactions

**DOI:** 10.1021/acsinfecdis.0c00552

**Published:** 2020-10-12

**Authors:** Serena Massari, Jenny Desantis, Maria Giulia Nizi, Violetta Cecchetti, Oriana Tabarrini

**Affiliations:** † Department of Pharmaceutical Sciences, University of Perugia, 06123 Perugia, Italy; ‡ Department of Chemistry, Biology and Biotechnology, University of Perugia, 06123, Perugia, Italy

**Keywords:** anti-influenza small molecules, RNA-dependent RNA polymerase, protein−protein interface inhibitors, PA−PB1, PB1−PB2, PB1−RanBP5, PB2−importin-α, PA−Pol II CTD, PB2−ANP32

## Abstract

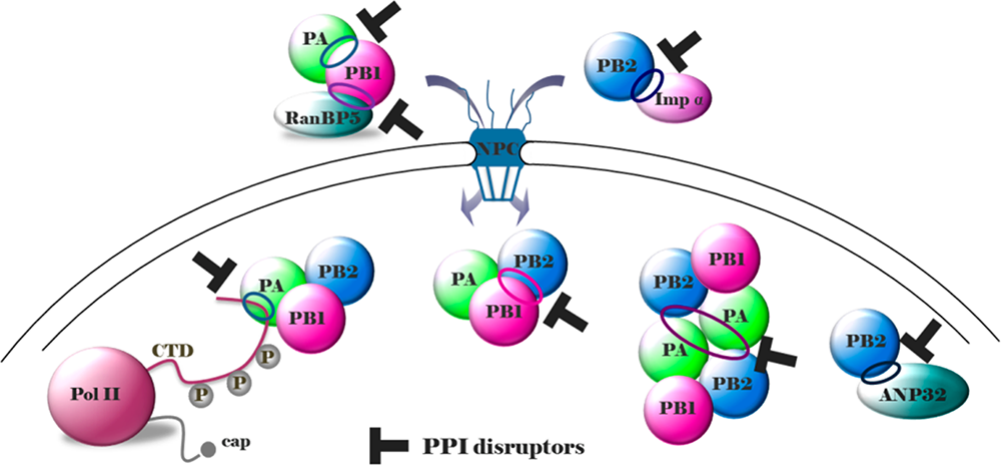

Influenza (flu) virus is a serious threat to global health with the potential to generate devastating pandemics. The availability of broad spectrum antiviral drugs is an unequaled weapon during pandemic events, especially when a vaccine is still not available. One of the most promising targets for the development of new antiflu therapeutics is the viral RNA-dependent RNA polymerase (RdRP). The assembly of the flu RdRP heterotrimeric complex from the individual polymerase acidic protein (PA), polymerase basic protein 1 (PB1), and polymerase basic protein 2 (PB2) subunits is a prerequisite for RdRP functions, such as mRNA synthesis and genome replication. In this Review, we report the known protein–protein interactions (PPIs) occurring by RdRP that could be disrupted by small molecules and analyze their benefits and drawbacks as drug targets. An overview of small molecules able to interfere with flu RdRP functions exploiting the PPI inhibition approach is described. In particular, an update on the most recent inhibitors targeting the well-consolidated RdRP PA–PB1 subunit heterodimerization is mainly reported, together with pioneer inhibitors targeting other virus–virus or virus–host interactions involving RdRP subunits. As demonstrated by the PA–PB1 interaction inhibitors discussed herein, the inhibition of flu RdRP functions by PPI disrupters clearly represents a valid means to identify compounds endowed with a broad spectrum of action and a reduced propensity to develop drug resistance, which are the main issues of antiviral drugs.

Viruses are able to generate pandemics with a devastating socio/economic impact in the world. In 1918, humanity witnessed the deadliest pandemic in human history, the Spanish flu, which caused extraordinary mortality around the globe.^1^ There is great concern that influenza (flu) viruses may cause another unpredictable devastating pandemic, perpetuated by the continuous emergence of new flu A strains.

Flu viruses are classified into four types (A, B, C, and D) on the basis of the highly conserved internal proteins matrix protein 1 (M1), membrane matrix protein (M2), and nucleoprotein (NP). On the basis of the surface proteins, hemagglutinin (HA), and the neuraminidase (NA), flu A viruses are further classified into different subtypes.^2^ Flu A and B viruses are relevant human respiratory pathogens, circulating among humans and causing seasonal epidemics. The generation of new epidemic strains occurs during the process of antigenic drift, in which the viral error-prone RNA-dependent RNA polymerase (RdRP) introduces mutations in the genes responsible for encoding the antigenic proteins HA and NA. On the other hand, new pandemic strains are generated during the process of antigenic shift, which occurs when two different flu A subtypes replicate within the same cell and blend segments of their genome, generating viral particles with a new reassortment of HA or NA antigens. This event may be responsible for severe pandemic outbreaks when highly virulent and pathogenic flu A strains cross the species barrier and acquire the ability to spread easily among human beings, which are naive to the novel strain.

Depending on the origin host, flu A viruses can be classified as avian flu, swine flu, or other types of animal flu viruses. Avian flu A viruses are classified as highly pathogenic avian influenza (HPAI) or low pathogenicity avian influenza (LPAI), on the basis of molecular characteristics of the virus and its ability to cause disease and mortality in chickens in a laboratory setting but not on the severity of illness in the cases of human infection.

In 2009, the swine flu A(H1N1)pdm09 generated the first pandemic of the XXI century with 500,000 infected people and 18,000 deaths.^3,4^ Currently, swine flu A subtypes A(H1N1)pdm09 and A(H3N2) and flu B lineages B/Yamagata and B/Victoria are circulating in humans, and quadrivalent influenza vaccines used for prevention contain their most recent strains.^5^

Of particular concern to public health are two avian flu A subtypes, the HPAI A(H5N1) and the LPAI A(H7N9).^6^ The first human infection by flu A(H5N1) was reported in 1997 in Hong Kong; then, it re-emerged in 2003 in China and, since then, sporadic human infections have been reported in several countries. Since 1996, it has caused more than 1000 deaths, with a mortality rate as high as 55%.^7^ In 2013, the flu A(H7N9) virus emerged in China, infecting humans and causing a severe respiratory disease with a high fatality rate (40%); to date, the flu A(H7N9) strain has infected over 1600 humans with 623 fatalities.^8^ Although there has been only rare evidence of a sustained human-to-human transmission of these avian flu strains, the possibility that they could change and gain the ability to spread easily between people poses a serious and constant threat to global public health.

On the basis of WHO data, 300,000 to 650,000 people die each year from all variants of the virus during seasonal epidemics in the world.^5^ Many researchers are working on a universal flu vaccine, meaning that a single injection would protect against all known and emerging flu A strains and last a lifetime.^9^ On the other hand, existing vaccines, which are effective against the flu causing an annual epidemic, must be updated each year since they are rendered ineffective by the major antigenic determinants of flu viruses.^10^ Moreover, they only are effective against specific flu strains; their effectiveness is variable within the host population, and the lag time needed to produce a new vaccine may be too long to fight a new pandemic.

Accordingly, antiviral drugs are greatly needed for the management of the flu pandemic, but the therapeutic armamentarium for prophylaxis and treatment of flu infections is very limited.^11^ After almost 20 years from their approval, the NA inhibitors oseltamivir and zanamivir remain the sole widely used drugs for clinical use.^12^ Indeed, emergence of widespread resistance has made M2 ion channel inhibitors no longer recommended,^13^ and the recently approved NA inhibitors peramivir and laninamivir octanoate have important limitations. In particular, common side effects of laninamivir include nausea, vomiting, diarrhea, dizziness, and decreased neutrophil count,^14,15^ while peramivir has a very low bioavailability, and thus, the compound is administrated only as an intravenous formulation.^16^ Moreover, although resistance to NA inhibitors is less frequent than that to adamantanes, isolates with reduced susceptibility to NA inhibitors were reported among avian strains. The limited antiflu therapeutic armamentarium has been recently enriched by compounds targeting the flu RdRP, such as favipiravir^17^ and baloxarir marboxil^18^ (major details are given below in the description of RdRP inhibitors).

In the search for next-generation flu antivirals, the RdRP has been validated as a superior antiviral drug target.^19−23^ It is essential for viral transcription and replication; its structure is highly conserved among all the flu strains, and its activity is highly host- and cell-type specific. The RdRP is a heterotrimeric complex composed of polymerase basic protein 1 (PB1, 757 aa in flu A), polymerase basic protein 2 (PB2, 759 aa), and polymerase acidic protein (PA, 716 aa, P3 in flu C) subunits, which extensively interact with each other in a tightly associated and coupled fashion.^24,25^ Their correct assembly is pivotal for RdRP activities, such as cap-binding, endonuclease, polymerase, and polyadenylation. RdRP works in the context of the viral ribonucleoprotein (vRNP) complex, in which each of the eight (seven for flu C and D) single-stranded negative-sense viral RNA (vRNA) segments is coated by multiple copies of the NP and bounded, at the 5′ and 3′ termini, to the viral RdRP.

In recent years, major progress has been made in revealing the structure and functions of the RdRP complex, not only furnishing significant insights into the molecular mechanism of transcription and replication but also creating unique opportunities for a structure-based drug design (SBDD) strategy. Readers are directed to recent reviews^24−29^ for a comprehensive discussion on flu RdRP structures and functions. Cusack and co-workers made an outstanding contribution by solving the crystal structure of the whole RdRP, determined from flu A/little yellow-shouldered bat/Guatemala/060/2010 (H17N10),^30^ flu B/Memphis/13/2003,^31^ and flu C/Johannesburg/1/1966^32^ strains. Transcriptionally active flu RdRP is U-shaped with the two upper protuberances being the PA_N_ endonuclease and the PB2_C_ cap-binding domains, the interior filled by the PB1 polymerase domain, and the bottom formed by the PA_C_ domain (Figure 1). The stable link between the subunits is mainly ensured by hydrophobic interfaces of PA_C_ with PB1_N_ and PB1_C_ with PB2_N_, while the high conformational flexibility of the heterotrimer is due to numerous intersubunit hydrophilic interactions.

**Figure 1:**
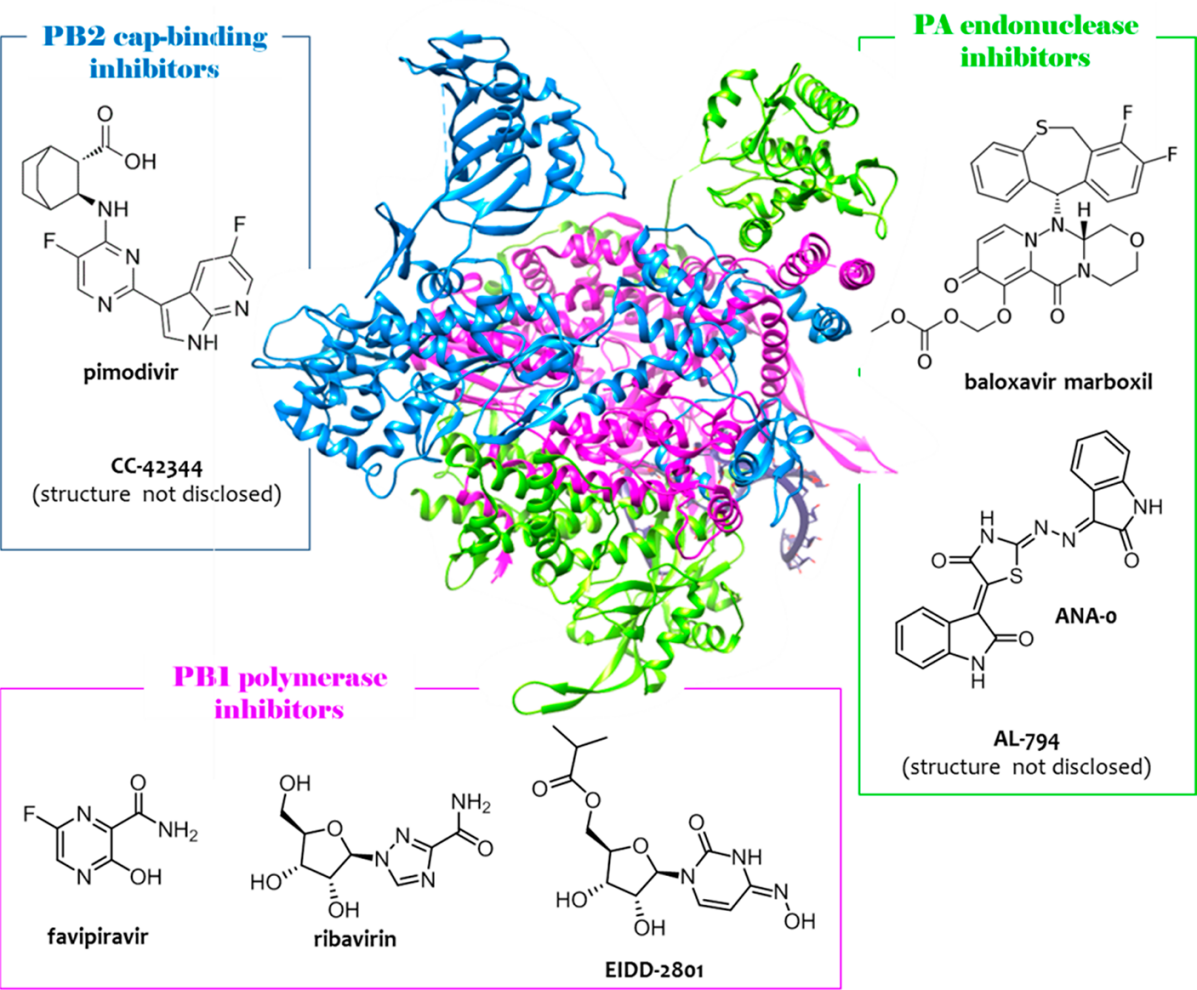
Crystal structure of flu A RdRP determined from bat flu A H17N10 strain (pdb: 4WSB(30)) and chemical structures of inhibitors of PA (green), PB2 (blue), and PB1 (magenta) subunits, approved or in the pipeline. The overall RdRP is U shaped with the PA_N_ endonuclease and PB2 cap-binding domains being the two upper protuberances, the PA_C_ domain being the bottom, and the PB1 polymerase domain filling the interior. Among the reported compounds, PA endonuclease inhibitor baloxavir marboxil and PB1 inhibitor favipiravir have been approved. The figure is author created, and the RdRP structure has been adapted from the pdb mentioned above and drawn by using UCSF Chimera package.^47^

Transcription is a primer-dependent process, in which the vRNA segments are used as template by the RdRP for generating 5′ capped and 3′ polyadenylated mRNA molecules. On the other hand, replication of vRNA segments is a two-step process, in which the vRNA is initially copied into a complementary RNA (cRNA), a replicative intermediate that, in the context of the complementary ribonucleoprotein (cRNP) complex, acts as the template for synthesis of vRNA. Very recently, Fodor and co-workers determined, by crystallography and cryo-electron microscopy, the structures of polymerase from human flu A/NT/60/1968 (H3N2) and avian flu A/duck/Fujian/01/2002 (H5N1) strains in the presence or absence of a cRNA or vRNA template,^33^ providing important insights into the replication mechanisms of the vRNA genome.

Although viral polymerase is one of the main drug targets for antivirals research, only recently there was a turning point in the development of flu RdRP inhibitors, with the identification of some very interesting compounds targeting each one of the three polymerase subunits.^34^ In particular, the nucleoside analog favipiravir (T-705 or avigan)^17^ was approved in 2014 in Japan; the PA endonuclease inhibitor baloxavir marboxil^18^ was approved in 2018 in both Japan and the USA, and the PB2 cap-binding inhibitor pimodivir^35^ has undergone phase 2b studies^36^ and advanced to phase 3 studies (Figure 1).

Other RdRP inhibitors worthy of note are PB1 inhibitors ribavirin^37,38^ and **EIDD-2801**,^39,40^ PA endonuclease inhibitors **AL-794**(41) (**JNJ-64155806**) and **ANA-0**,^42^ and the PB2 inhibitor **CC-42344** (Figure 1). Ribavirin is a guanosine analogue approved in 1986 as a broad-spectrum antiviral drug. It was developed as an antiflu agent on the basis of its efficacy in a mouse model of influenza,^43,44^ but its effect in human clinical trials was less clear; thus, it was not approved for the treatment of influenza.^45^ Compound **EIDD-2801**(39) is a prodrug showing good oral bioavailability, broad-spectrum inhibition of seasonal and highly pathogenic flu A and B viruses, and a high barrier against resistance; efficacy testing using a ferret model of flu infection demonstrated low toxicity and potent efficacy of the compound.^40^ Compound **AL-794**(41) (structure not disclosed) was discovered by Alios Biopharma; although its oral administration showed a significant dose-dependent antiviral activity and a good safety profile, its development has been discontinued as early phase 1 studies identified the inability to establish a single safe effective dose across all patients. Compound **ANA-0** is currently under preclinical evaluation; it has been identified by Yuan et al.^42^ through a screening approach and showed broad and potent antiflu activity. Compound **CC-42344** was developed by Cocrystal Pharma and is currently being evaluated in preclinical IND-enabling studies for the treatment of influenza. **CC-42344** (structure not disclosed) exhibited broad and potent antiviral activity (IC_50_ ranging from 0.1 to 9 nM) against a panel of seasonal and pandemic flu A strains using *in vitro* cytopathic effect inhibition assays. The discovery and *in vitro* characterization of **CC-42344** have not yet been disclosed, while preclinical pharmacokinetic and safety profiles were reported as favorable.^46^

The approval of RdRP inhibitors confirmed the high profile of the RdRP as drug target, although they suffer from some limitations. In particular, baloxavir marboxil and pimodivir led to the rapid development of resistant viruses *in vitro*(48,49) and are characterized by a narrow range of antiviral activity, with pimodivir that inhibits only flu A strains and baloxavir marboxil that inhibits also flu B strains but at higher concentrations.^35,49^ Pimodivir is a very potent inhibitor of flu A RdRP with picomolar affinity for the PB2 cap-binding site,^35^ while it binds weakly to the flu B cap-binding domain due to amino acids differences in the cap-binding sites among flu A and B; the major loss in affinity derives from the substitution of Q325 in flu B instead of F323 in flu A, which impairs a strong π-stacking with the pyrimidine ring of the pimodivir.^50^ On the contrary, the broad spectrum of activity shown by baloxavir marboxil could be explained by high conservation of amino acids forming the cap-dependent endonuclease resides in the PA subunit across seasonal, pandemic, and highly pathogenic avian influenza viruses.^49^ Analogously, the highly conserved catalytic polymerase domain among various types of RNA viruses could justify the broad spectrum of antiviral activity shown by favipiravir.^51,52^ Moreover, attempts to select escape mutants to favipiravir failed due to its ability to induce lethal mutagenesis. Nevertheless, it is characterized by unfavorable pharmacokinetics, high loading doses, and teratogenic effects.^34,53^

An emerging approach to develop compounds with a high barrier to drug resistance is the inhibition of the RdRP functions by interfering with protein–protein interactions (PPIs) among RdRP subunits. The main potential advantages of targeting PPIs are (i) the great variability and specificity of PPIs with respect to the active site of an enzyme, (ii) their high degree of conservation among the different strains, and (iii) the requirement for the simultaneous mutation of at least one residue on both proteins involved in the interaction to develop resistance. Accordingly, PPI inhibitors could show broad-spectrum antiflu activity and a high barrier to drug resistance, thus overcoming the main limitations that characterize the currently available treatment.

This Review reports on the current status of the small molecules that interfere with flu RdRP functions by inhibiting one of its PPIs. An analysis of the known interactions occurring by RdRP subunits will be initially given, focusing on those already targeted by small molecules but also those for which the crystal structure is available and thus could serve as alternative drug targets. Then, an update of the most recent inhibitors targeting the well-consolidated RdRP PA–PB1 heterodimerization will constitute the main body of the work, along with pioneer inhibitors targeting other virus–virus and virus–host interactions by RdRP subunits; approaches used for their identification, the hit-to-lead studies, the structure–activity relationship (SAR) insights, and the hypothesized binding modes will be described.

## Protein–Protein Interactions by RdRP Subunits as Drug Targets

Through interactions with multiple host factors, the vRNP components play vital roles in replication, host adaptation, interspecies transmission, and pathogenicity.^54,55^ When one focuses on RdRP subunits, multiple PPIs are established not only among themselves but also with a number of host proteins that are essential cofactors for RdRP localization and functions.

A detailed discussion on the wide variety of host proteins hijacked during flu virus replication is beyond the scope of this work, and readers are referred to comprehensive reviews^54,55^ on this topic. Following, we will focus only on those interactions occurring by RdRP subunits, both among themselves and with host factors that could serve or have already been employed as alternative antiflu targets, enumerated on the basis of the steps of the RdRP journey.

**Figure 2:**
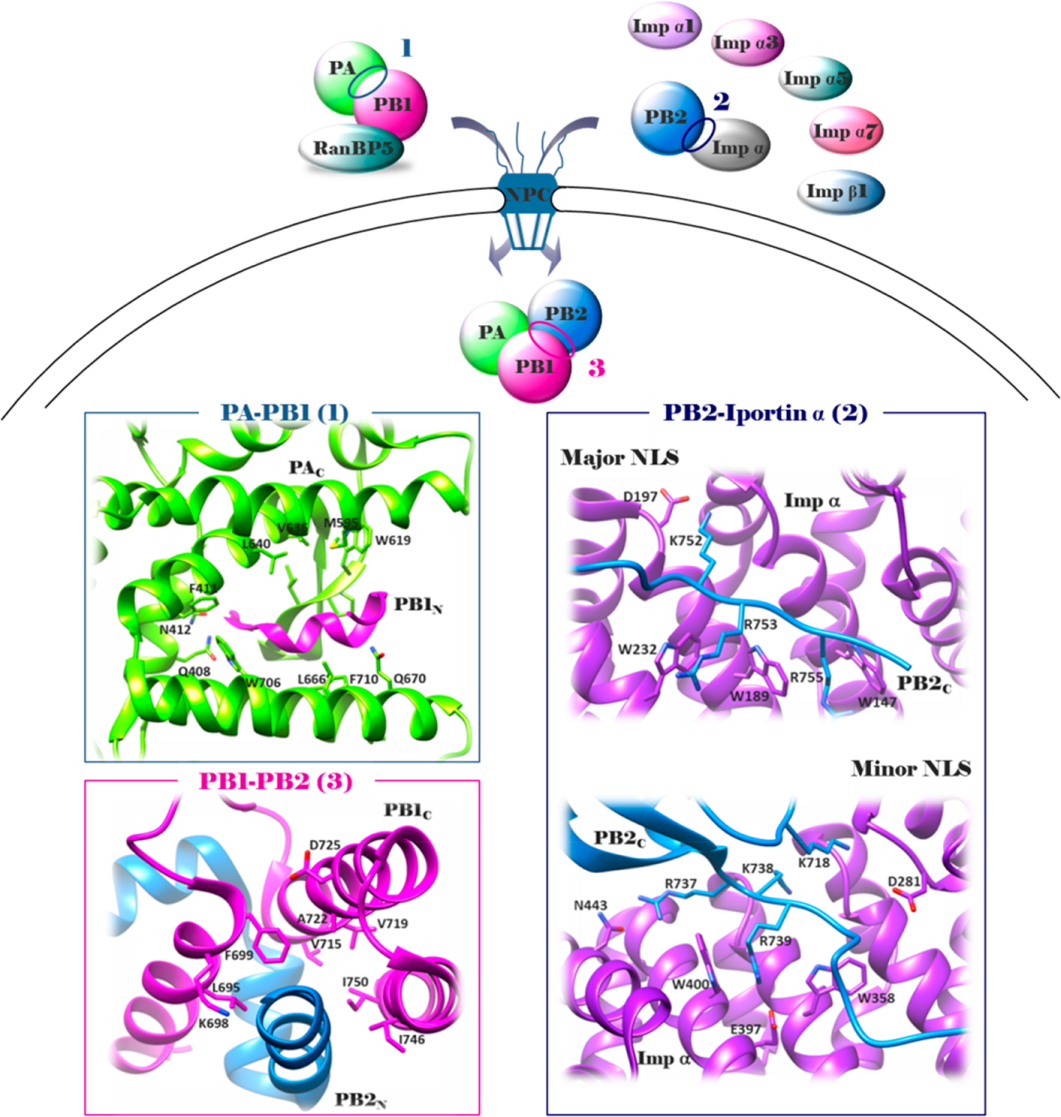
Schematic representation of flu RdRP subunit nuclear localization and heterotrimerization (upper side): in the cytoplasm, PA and PB1 form a heterodimer (extensive interactions occurring between PA_C_ and PB1_N_); then, the PA–PB1 heterodimer associates with RanBP5 (interaction occurring at the PB1_N_ bipartite NLS), and PB2 associates with importin-α1, -α3, -α5, or -α7 (interaction occurring at the PB2 NLS), which then binds to importin-β1, to enter within the nucleus; finally, once in the nucleus, PA–PB1 and PB2 associate (extensive interactions occurring between PB1_C_ and PB2_N_) to form the whole RdRP heterotrimer. For clarity, the RdRP is shown alone and not in the context of the vRNP. Crystal structures of PA–PB1 (pdb: 3CM8(72)), PB2–importin-α (as an example, the PB2–importin-α7 complex was shown; pdb: 4UAD(61)), and PB1–PB2 (pdb: 2ZTT(76)) interfaces (lower side). PA subunit, green; PB2 subunit, blue; PB1 subunit, magenta. The figure is author created, and the structures have been adapted from the pdb mentioned above and drawn by using the UCSF Chimera package.^47^

*Nuclear localization* of the RdRP occurs through interactions of its subunits with importin-like factors and components of the nuclear pore complex (NPC) (Figure 2). In particular, PA and PB1 subunits enter within the nucleus as heterodimer through their further complexion with the host nuclear import factor Ran-binding protein 5 (RanBP5),^56^ a member of the importin-β superfamily. RanBP5 binds to the PB1–PA dimer through PB1. Although the crystal structure of the PB1–RanBP5 interface is not available, it is known that the RanBP5 binding site is located in the PB1_N_ at the level of the bipartite nuclear localization signal (NLS) motifs (NSL1, residues 187–190; NSL2, residues 207–211).^57^ Notably, PB1_N_ mutations affecting RanBP5 binding (single or double mutations at residues 188–189 and 208–209) severely attenuated or were incompatible with viral growth, although did not entirely prevent PB1 nuclear accumulation. PB1_N_ residues involved in the RanBP5 binding are conserved across a broad range of flu A strains and are unlikely to be a determinant of host tropism.^57^

Nuclear localization of PB2 depends on its association with importin-α1, -α3, -α5, or -α7, which then binds to importin-β1.^58^ Besides mediating PB2 nuclear import, importin-α seem to have a role in viral transcription and replication.^59,60^ In particular, mutations of PB2 at the level of the importin-α binding site greatly impaired polymerase activity, while showing only a modest reduction in PB2 nuclear accumulation.^59^ Crystal structures are available for PB2–NLS (res. 678–759) in complex with each of the importin-α isoforms (pdb: 4UAF for PB2–importin-α1;^61^4UAE for PB2–importin-α3;^61^2JDQ for PB2–importin-α5;^60^4UAD for PB2–importin-α7^61^). All importin-α isoforms share an essentially invariant NLS-binding surface, although differ greatly in conformational flexibility. PB2_C_ residues 678–736 sit above the minor NLS-binding pocket of importin-α while PB2_C_ residues 737–759 make extensive contacts that span from minor to major NLS-binding pockets (Figure 2). Of note, the domain contains two surface residues, D701 and R702, implicated in the adaptation from avian to mammalian hosts.^62^ Residue 701 is always an aspartate in all flu strains, but D701N adaptive mutation occurs in mammalian adapted flu strains;^63,64^ additionally, residue 702 is an arginine in human isolates and a lysine in avian strains.^65^ Although the exact role of the two residues and their adaptive mutation remain to be elucidated, it has been suggested that PB2 mutations affect both its interaction with importin-α and importin-α usage. Thus, PB2 of avian flu viruses uses importin-α3 in human cells, while PB2 of mammalian-adapted flu viruses uses importin-α7.^66,67^

In an alternative model, PB1 and PB2 form a heterodimer at the cytoplasmic level and enter the nucleus via complexation with the heat shock protein 90 (Hsp90).^68^ The interaction between Hsp90 and PB2 can be mapped to the middle and N-terminal domains of Hsp90 and the N-terminal or middle portion of PB2.

Blocking nuclear localization of flu RdRP subunits is a potentially promising mechanism for new antivirals, and attempts have been already made, although they mainly focused on targeting host factors and not their interaction with RdRP subunits. Chase et al. reported that inhibition of Hsp90 by geldanamycin and 17-allylamino-demethoxygeldanamycin impairs flu viral growth and delays the accumulation of mRNA, cRNA and vRNA, although no significant difference in trimeric RdRP levels was detected.^69^ Resa-Infante et al. analyzed the feasibility of targeting importin-α7 in an *in vivo* animal model, but pandemic H1N1 flu viruses were able to escape the requirement for importin-α7 by acquiring adaptive mutations in the vRNP and surface glycoproteins, which rendered the virus even more virulent.^70^ On the other hand, Mohl et al. reported the first small molecules impairing PA–PB1 nuclear localization by interfering with the PB1–RanBP5 interaction^71^ (see PB1–RanBP5 Inhibitors).

As reported above, *PA–PB1 heterodimerization* occurs in the cytoplasm, while once shuttled at the nuclear level, the PA–PB1 complex and PB2 dissociate from their import factors and assemble to form the heterotrimer (Figure 2). Thanks to its heterotrimeric structure, flu RdRP itself is particularly suitable for exploiting the PPI inhibition approach. The three RdRP subunits are stably linked in head-to-tail fashion by extensive interactions occurring between the PB1_N_ and PA_C_ termini and the PB1_C_ and PB2_N_ termini. Crystal structures of both the PA_C_–PB1_N_ and PB1_C_–PB2_N_ interfaces have been reported in 2008^72,74^ and 2009,^76^ respectively.

He et al. published the structure of PA_C_ (residues 257–716) in complex with the 25 PB1_N_ peptide from the avian flu A/goose/Guangdong/1/1996 (H5N1) strain (pdb: 3CM8, Figure 2).^72^ PA_C_ resembles the head of a dragon, of which the brain is domain I and the mouth, domain II. PB1_N_ mainly interacts with a hydrophobic core (defined by four α-helices) of PA_C_, thus establishing largely hydrophobic interactions but also H-bonds and van der Waals forces. A successive molecular dynamic study carried out by Liu and Yao on this structure identified three pockets within the PA_C_ hydrophobic core: the first defined by W706 and F411 involved in the interaction with PB1_N_ P5, the second defined by F710 and L666 involved in the binding with PB1_N_ F9, and the third pocket defined by L640, V636, M595, and W619 involved in the interaction with PB1_N_ L8.^73^ The crystal structure indicated additional interactions between the PB1_N_ D2-A14 motif and residues of the PA_C_ hydrophobic core, such as Q408, N412, Q670, P620, and I621.^72^ The double mutation of residues of the PA_C_ hydrophobic core disrupted the PA_C_–PB1_N_ binding.

In parallel, Obayashi et al. reported the crystal structure of PA_C_ (residues 239–716) in complex with the PB1_N_ terminal 81 residues from the flu A/Puerto Rico/8/1934 (H1N1) strain (pdb: 2ZNL).^74^ The structure confirmed the hydrophobic nature of PA_C_, with hydrophobic interactions contributing substantially to the PA_C_–PB1_N_ binding, although also numerous H-bonds are present. Moreover, the structure highlighted additional PA_C_ residues, such as E617, T618, E623, and R673, which are involved in the binding with PB1_N_ (residues K11, D2, and L10) thorough H-bonds. Mutations V636S, L640D, and W706A of PA_C_ greatly weakened or abolished PB1_N_ binding and reduced the synthesis of vRNA, cRNA, and mRNA.

Thus, both structures suggested that the PA_C_–PB1_N_ interface is characterized by relatively few residues driving the subunit binding, suggesting the feasibility to use small molecules to interfere with this PPI. Moreover, the first 15 PB1_N_ residues, which are involved in the PA_C_ binding, are completely conserved among avian and human flu strains.^75^

Crystal structures of PB1_C_–PB2_N_ (PB1_C_: residues 678–757; PB2_N_: residues 1–37) (pdb: 2ZTT and 3A1G; Figure 2)^76^ from the flu A/Puerto Rico/8/1934 (H1N1) strain showed that, unlike the interaction between the PA_C_ and PB1_N_ that has a predominantly hydrophobic character, the PB1_C_–PB2_N_ interface is characterized by more polar interactions and is more extensive in sequence length and buried surface area (1400 Å^2^). The interface area includes four salt bridges (three between K698 and E2, R3, and E6 and one between D725 and R3) and eight H-bonds between the polypeptides involving main-chain atoms. The majority of the interaction energy appears to be contributed by PB2_N_ helix 1, which involves four salt bridges to PB1_C_ and the key apolar contacts, such as those with I4 and L7. Functional studies confirmed the importance of helix 1 of PB2_N_ to vRNA synthesis, as deletion of this helix (residues 1–12) greatly reduces the RdRP activity.^76^ The mutation of key PB2_N_ residues also showed a dramatic reduction in mRNA synthesis with various interface mutants, such as L7D that impairs PB1_C_–PB2_N_ binding.^76^ On the other hand, some of the PB1_C_ mutants showed very different effects on PB1_C_–PB2_N_ binding and RdRP activity, such as F699A and I750D mutants showing weak PB2 binding but increased enzyme activity. These results showed that, although small, the PB1_C_–PB2_N_ interface has a crucial function not only in the RdRP subunit interaction but also in regulating the whole RdRP complex. Moreover, residues of both PB1_C_ and PB2_N_ involved in the binding are completely conserved among avian and human flu viruses.

On the basis of the structural features, both PA_C_–PB1_N_ and PB1_C_–PB2_N_ interfaces appear to be suitable for drug design. Nevertheless, to date, only one class of small molecules has been reported as PB1–PB2 inhibitors^77^ (see PB1–PB2 Inhibitors), while since 2012, more intense efforts have been devoted to identify PA–PB1 interaction inhibitors with interesting compounds that continue to appear in the literature^78^ (see PA–PB1 Interaction Inhibitors).

**Figure 3:**
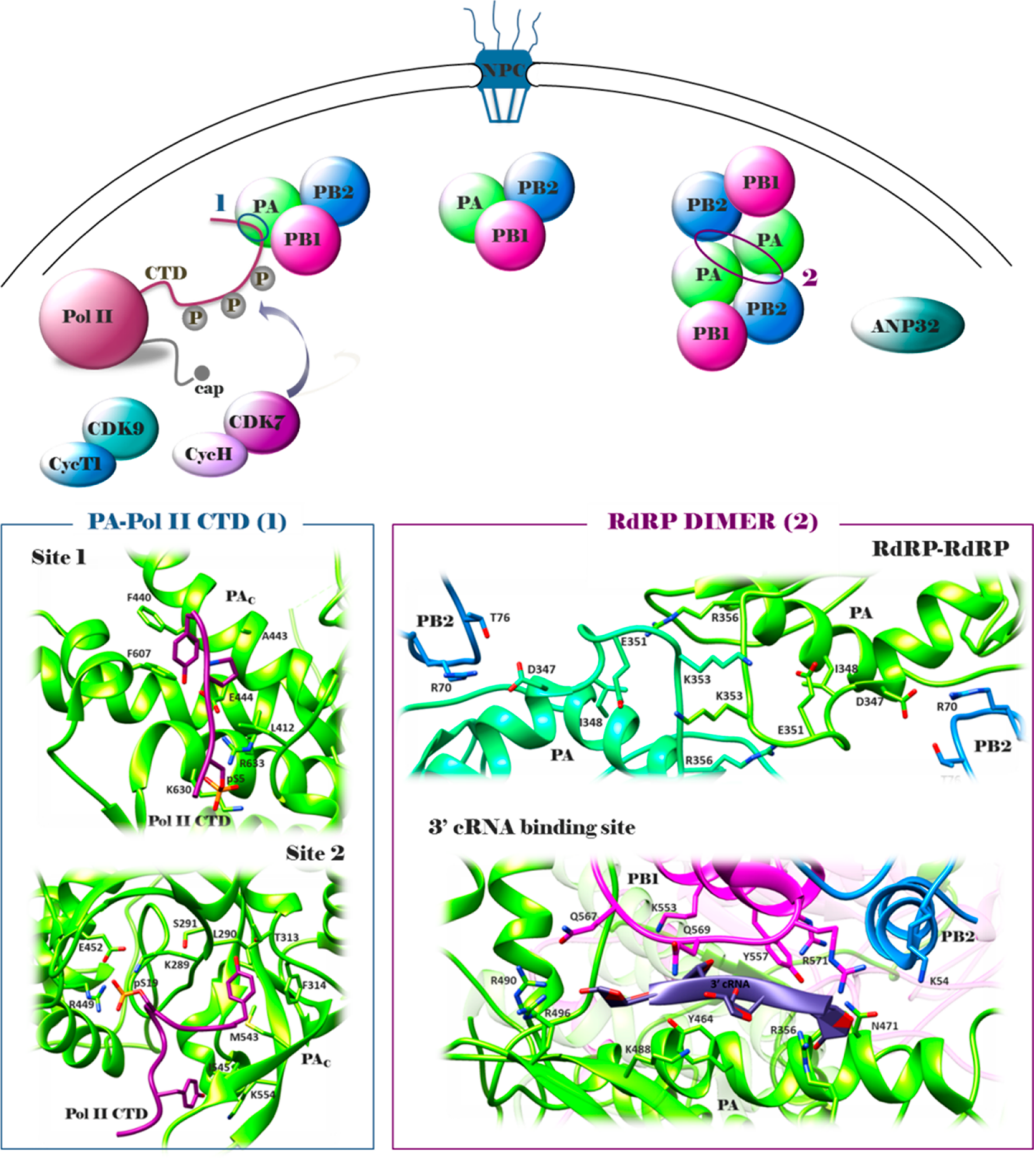
Schematic representation of the flu RdRP association with Pol II during vRNA transcription and dimerization during vRNA replication (upper side): once heterotrimerization has occurred in the nucleus, RdRP performs both the processes of transcription and replication; during transcription of viral mRNA, the specific interaction between the PA_C_ and host Pol II CTD is required to enable the process of cap-snatching; during replication, a new RdRP is synthesized and associates with the resident RdRP to form a dimer, which is required for the synthesis of cRNA from vRNA (interactions occurring between the PA_C_ loop of the two RdRPs and PA_C_ of one RdRP and PB2 loop of the other); finally, the association of PB2 and host factor ANP32 promote the replication of vRNA from cRNA (interaction occurring at the PB2 627 domain). For clarity, the RdRP is shown alone and not in the context of the vRNP. Crystal structures of the PA–Pol II CTD interface (pdb: 5M3H(81)), RdRP–RdRP interface (pdb: 6QPG(33)), and 3′ cRNA binding site (pdb: 6QX3(33)) (lower side). PA subunit, green; PB2 subunit, blue; PB1 subunit, magenta. The figure is author created, and the structures have been adapted from the pdb mentioned above and drawn by using the UCSF Chimera package.^47^

One of the best known factors involved during *transcription* of viral mRNA by the RdRP is the host RNA polymerase II (Pol II) (Figure 3). The specific interaction between the RdRP PA subunit and Pol II carboxy-terminal domain (CTD)^79^ is required to enable the process of “cap-snatching”, in which the flu RdRP takes short capped oligomers from nascent Pol II transcripts to be used as transcription primers. The association between RdRP and Pol II CTD occurs when Pol II CTD, consisting of 52 heptad repeats (Y_1_S_2_P_3_T_4_S_5_P_6_S_7_), is phosphorylated on Ser-5 by CDK7 (in complex with CycH to form TFIIH) but not yet on Ser-2 by CDK9 (in complex with CycT1 to form P-TEFb).^80^ Crystal structures of RdRP from flu A/little yellow-shouldered bat/Guatemala/060/2010 (H17N10) (pdb: 5M3H; Figure 3) and flu B/Memphis/13/2003 (pdb: 5M3J) bound to vRNA and a four-heptad repeat phosphorylated Ser-5 have been recently determined.^81^ In particular, six residues from one CTD repeat are accommodated in a PA_C_ phosphoserine binding site (site 1, interaction surface of 672 Å, key aa involved in phosphate binding K653 and R638), and ten residues from three consecutive repeats are accommodated in a second PA_C_ phosphoserine binding site (site 2, interaction surface of 1168 Å, key aa involved in phosphate binding K289 and R454). Site 1 is conserved in all flu A and B but not flu C or D strains, while site 2 is only conserved in flu A strains. The recent publication of the crystal structure of RdRP from the flu C/Johannesburg/1/1966 strain in complex with the Pol II CTD (pdb: 6F5P)^82^ confirmed that its phosphorylated CTD binding sites are distinct from those of flu A and B RdRP. A phosphorylated CTD peptide with four heptad repeats showed *K*_d_ values of 0.9 μM for the RdRP–promoter complex, which decreased to *K*_d_ values of 3.6 and 6.9 μM when the affinity was evaluated by using bat flu A RdRP with double mutants in site 1 or site 2, respectively.^81^ Accordingly, the minigenome assay showed a marked decrease in overall RdRP activity when double mutated in each site, and RT-PCR analysis suggested a strong decrease in mRNA levels.^81^

A further PPI that could be investigated for the development of innovative antiflu agents is that between two RdRPs occurring during genomic vRNA *replication*. In particular, during the synthesis of cRNA from vRNA by the RdRP, the nascent cRNA assembles into a cRNP complex with NPs and a newly synthesized RdRP. In this context, the resident and the newly synthesized RdRPs form a **dimer** that is required for the initiation of vRNA synthesis on the cRNA template. Recently, structures of the complete RdRP from human flu A/NT/60/1968 (H3N2) and avian flu A/duck/Fujian/01/2002 (H5N1) strains have been reported (crystal structures pdb: 6QNW for flu A(H3N2) RdRP; 6QPF for flu A(H5Nl) RdRP; 6QPG for flu A(H3N2) RdRP–Nb8205; Cryo-EM structures pdb: 6QX8 for dimeric flu A(H3N2) RdRP–cRNA; 6QWL for monomeric flu B RdRP–cRNA; 6RR7 for monomeric flu A(H3N2) RdRP–vRNA–capped RNA; 6QX3 for monomeric flu A(H3N2) RdRP–cRNA–Nb8205; 6QXE for dimeric flu A(H3N2)–cRNA–Nb8205)^33^ (Figure 3). The structures suggested that, in solution, flu A RdRP forms dimers of heterotrimers through all the three subunits, PB1 thumb and PB2 N1 subdomains and the PA_C_ domain. In particular, interactions occur between the PA_C_ loop 352–356 and the same loop of the second polymerase as well as H-bonds between the PA_C_ residue D347 of each polymerase and PB2 loop 71–76 of the other one. The mutation of PA_C_ loop residues resulted in a shift toward a monomeric heterotrimer. Besides the RdRP–RdRP interface that could serve as a drug target for the development of dimerization inhibitors, the structure of monomeric flu A RdRP bound to the cRNA template also revealed a binding site for the 3′ cRNA at the dimer interface that could be exploited for drug design. Interference at this dimer interface by a nanobody (Nb8205) inhibited flu RdRP dimerization, vRNA synthesis, and flu replication in infected cells. Also, in this case, numerous avian to mammalian adaptive mutations have been observed at the residues involved in the dimer interface, indicating that RdRP dimerization may be regulated in a host-specific manner.

Another association by RdRP that is known to be essential for its activity is with host acidic nuclear phosphoprotein 32 (ANP32) members A and B. In particular, the 627 domain of PB2 (aa 538–680) has been reported to interact in the nucleus of host cells with ANP32A and ANP32B,^83^ specifically promoting the replication of vRNA from cRNA.^84^ Simultaneous knockout of ANP32A/B in human cells abolished flu RdRP activity in the minigenome assay as well as viral growth.^85,86^ Of note, as already highlighted for the PB2 cross-species-transfer residues D701 and R702, residue 627 of PB2 is strongly implicated in host adaptation.^87^ It is almost invariably a glutamate in avian strains, but E627 K adaptive mutation is required for efficient polymerase activity in mammalian-adapted strains.^88^ This adaptive mutation has been correlated to characteristic differences between avian and human ANP32. To date, the molecular details of how ANP32 interacts with RdRP and the E627 K mutation allows the RdRP to work with mammalian ANP32 remain unknown.

In summary, RdRP subunits are involved in numerous PPIs. Some of the them have already been validated as drug target, such as PA–PB1, PB1–PB2, and PB1–RanBP5 interfaces, with small molecules that have been reported to successfully inhibit flu growth. For other interfaces, such as PA–Pol II CTD and RdRP–RdRP dimer, although not exploited yet, the proof-of-concept that RdRP functions could be blocked by their specific inhibition has already been provided by using a peptide and a nanobody, respectively. Moreover, the availability of the interface crystal structures offered the opportunity for SBDD. For other interactions involving host factors such as those between PB2 and importin-α and ANP32, although they are crucial for RdRP localization and/or functions, further studies are required to determine their potential as a drug target. In particular, the presence of cross-species-transfer residues in the PB2 domain involved in both the PPIs,^89,90^ of which the exact role remains to be elucidated, could limit their inhibition. For example, the nature of residue 627 has been linked with a dependency on avian and human ANP32 as well as on specific importin-α family members.^24^ Nevertheless, the complete map of amino acid mutations to the avian flu PB2 that enhance growth in human cells and, in particular, the examination of differential selection at known PB2 molecular interfaces such as with importin-α and Pol II CTD indicated that host-adaptive mutations are located adjacent to but not at core residues that directly interact with host proteins. These data suggested that host adaptation may involve mutations at sites at the periphery of core interactions.^91^

## Small Molecule Inhibitors of Protein–Protein Interactions by RdRP Subunits

### PA–PB1 Interaction Inhibitors

The PA–PB1 interface is the most studied and exploited among the PPIs by the RdRP subunits. A lot of evidence supports the validity of the PA–PB1 interface as the drug target. As already reported above, the interface is relatively small with few but highly conserved residues that drive the binding of PB1_N_ to PA_C,_ and it is largely hydrophobic, implying that it can be suitable for small molecule-mediated inhibition. Additionally, only a few substitutions of the key residues of the PA–PB1 interface were tolerated without a loss of binding, and such mutations resulted in severe impairment of RdRP functions and attenuation of viral replication.^92^ Additionally, a limited ability of flu viruses to compensate for mutations deliberately introduced into the PA_C_ or PB1_N_ termini was observed, indicating that escape mutations in these domains are a rare occurrence.^92^

**Figure 4:**
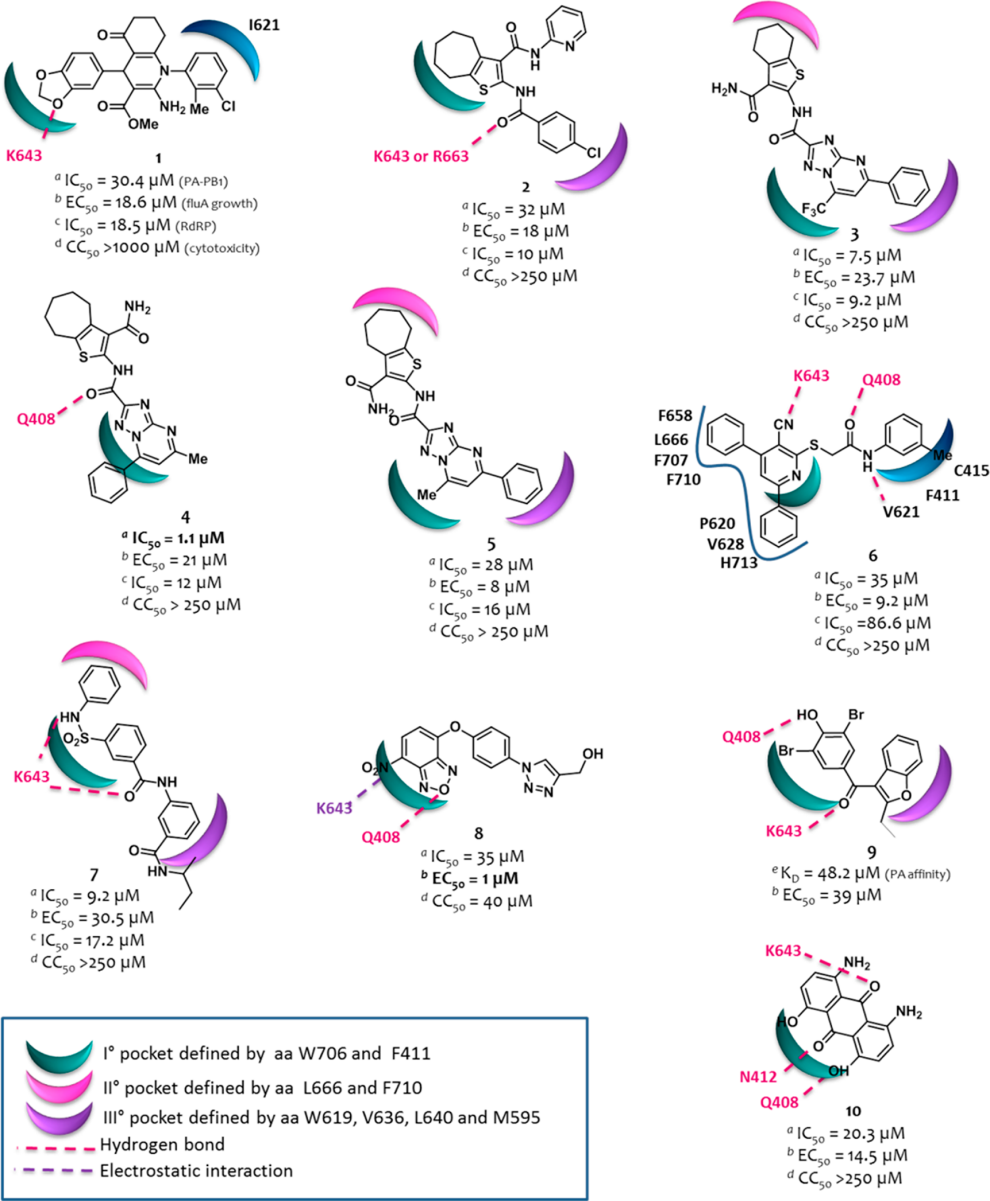
Structures of representative compounds reported as PA–PB1 inhibitors until 2015. ^*a*^The IC_50_ value represents the compound concentration that reduces the PA–PB1 complex formation by 50% (ELISA assay); ^*b*^the EC_50_ value represents the compound concentration that inhibits 50% of flu A replication (PRA assay); ^*c*^the IC_50_ value represents the compound concentration that reduces by 50% the activity of flu A virus RNA polymerase (minireplicon assay); ^*d*^the CC_50_ value represents the compound concentration that inhibits 50% of cell growth (MTT assay); ^*e*^the *K*_d_ value represents the dissociation constant of the compound with the PA cavity. The predicted binding mode of all the molecules, with the exception of **6**, in the PA cavity from structure 3CM8 was generated using FLAP.^78^ The predicted binding mode of **6** in the PA cavity (generated by Glide and GOLD) was reported as in the original paper.^99^ The figure is author created, while the original figures reporting the binding pose of compounds **1**–**10** are reported in Figures S1 and S2.

Since 2007, Schwemmle and co-workers have been pioneers in the field of PA–PB1 complex formation inhibitions, furnishing evidence that vRNA synthesis could be blocked by the specific inhibition of the RdRP PA–PB1 subunit interaction using small peptides.^75,93,94^ In 2008, the publication of the X-ray crystal structure of the PA–PB1 complex facilitated and prompted the discovery of the first small molecule inhibitors of this PPI, which appeared in the literature in 2012. Other compounds were successively identified, and those reported until 2015 were collected by us in a perspective,^78^ of which representative examples are shown in Figure 4, together with their biological activities, cytotoxicity, and hypothesized binding mode within the PA_C_ cavity (the original figures reporting the binding pose of compounds **1**–**10** are reported in Figures S1 and S2).

Some compounds have been identified by SBDD, such as compound **1**,^95^ while many of the others emerged by hit-to-lead optimization campaigns, such as cycloheptathiophene–3-carboxamide derivative **2**,^96^ triazolopyrimidine–2-carboxamide derivatives **3**,^97^**4**,^98^ and **5**,^98^ and pyridine derivative **6**.^99^ Other approaches, such as scaffold hopping, high throughput screening (HTS), and drug repurposing, led to identify compounds **7**,^97^**8**,^100^ and **9**,^101^ respectively, while compound **10**(102) was identified serendipitously.

With an IC_50_ of 1.1 μM, compound **4** is one of the most potent PA–PB1 heterodimerization inhibitors developed so far. This ability also translated to good antipolymerase and broad anti-flu A and B activities without showing any cytotoxicity up to the concentrations of 250 μM. The best antiflu activity was instead shown by benzofurazan derivative **8**, although endowed with a certain toxicity.

We recently analyzed the binding mode of all the best compounds reported until 2015 by a common approach (Figure 4) and also generated a pharmacophore model.^78^ The pharmacophore is quite planar and consists of two hydrophobic moieties, one of which is involved in the interaction with W706, a residue belonging to the first pocket defined by Liu and Yao^73^ (indicated in green in Figure 4). A polar belt with two H-bond acceptor points and one H-bond donor point separates the hydrophobic moieties. Large molecules usually exploit additional hydrophobic interactions with the second and/or third pockets defined by Liu and Yao^73^ (indicated in magenta and violet, respectively, in Figure 4). On the other hand, for small molecules unable to establish additional hydrophobic interactions, a favorable stabilization in the PA cavity is ensured by the formation of H-bonds, mainly with Q408 and K643.

The search for novel PA–PB1 interaction inhibitors has become increasingly active in the last years, with the identification of interesting compounds that are summarized in this Review.

**Figure 5:**
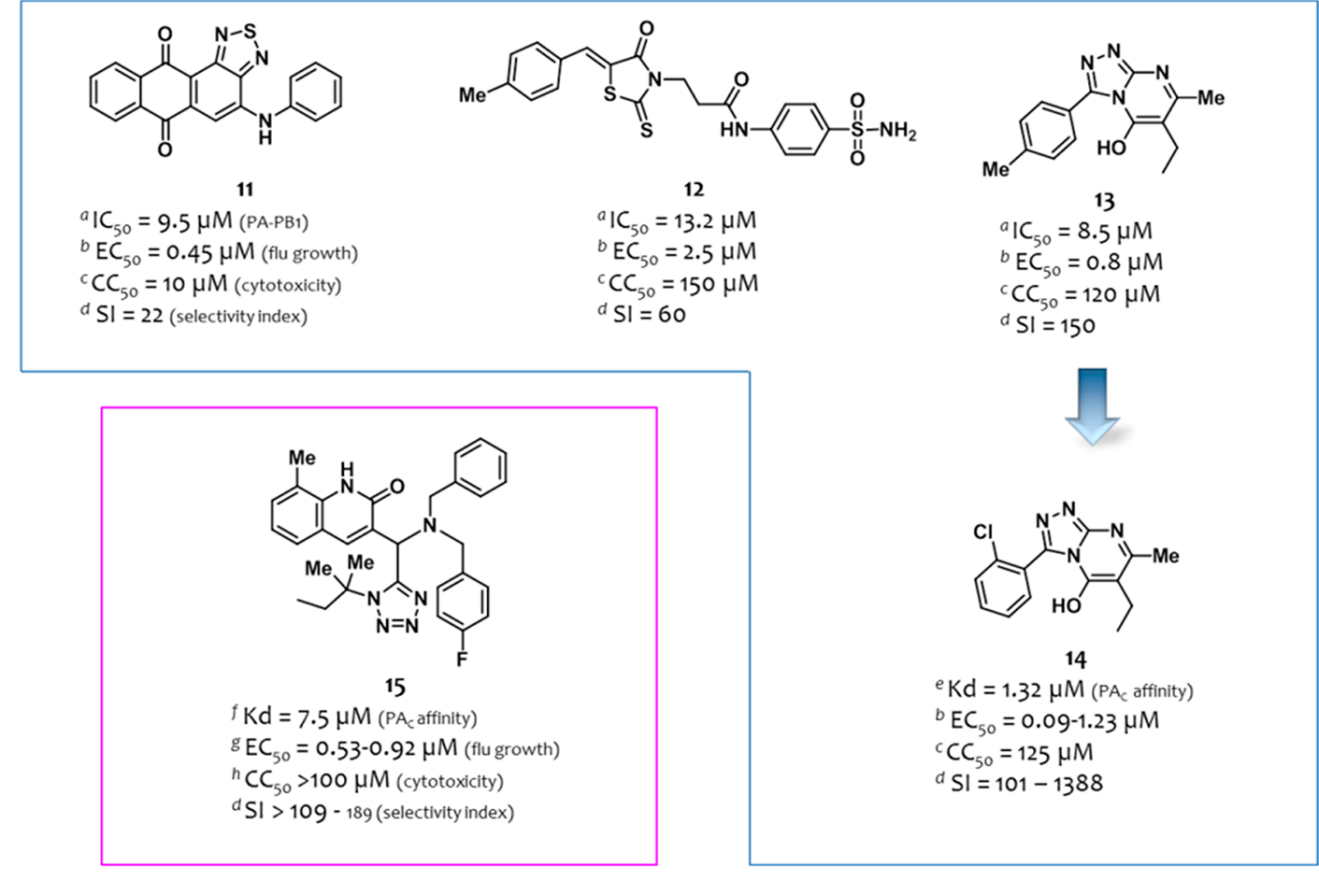
Structures and activities of PA–PB1 interaction inhibitors identified by Yuan et al.^103^ (in the blue box) and Watanabe et al.^104^ (in the magenta box). For the definition of IC_50_, EC_50_, CC_50_, and *K*_d_, see the Figure 4 caption. ^*a*^IC_50_, ELISA assay; ^*b*^EC_50_, PRA assay (MDCK cells); ^*c*^CC_50_, MTT assay (MDCK cell); ^*d*^SI (selectivity index) represents the ratio between CC_50_ and the highest/lowest EC_50_ values; ^*e*^K_d_, ITC assay; ^*f*^K_d_, SPR assay; ^*g*^EC_50_ and ^*h*^CC_50_, CV assay (MDCK cells). The figure is author created, while the original figures reporting the binding pose of compounds **14** and **15** are reported in Figures S3 and S4.

In 2016, after comparing the enzyme-linked immunosorbent assay (ELISA) and fluorescence polarization assay for the screening of PA–PB1 interaction inhibitors, Yuan et al.^103^ selected the ELISA assay to screen a library of 950 compounds, which were tested at the concentration of 10 μg/mL. The 27 compounds that showed a >50% decrease of binding intensity were then evaluated in a dose–response analysis, leading to the identification of 15 derivatives that consistently inhibited the PA–PB1 interaction with IC_50_ values < 2.5 μg/mL. Compounds were then evaluated in a secondary cell-based screening by the plaque reduction assay (PRA) (MDCK cells infected with the flu A/HK/415742/2009 (H1N1) strain), leading to the identification of compounds **11**–**13** (Figure 5) that showed dose-dependent antiflu activity. On the basis of their activity, cytotoxicity, and structural properties, 11 analogues with predicted good water solubility and low molecular weight were then synthesized. Among them, 1,2,4-triazolo[4,3-*a*]pyrimidin-5-ol derivative **14** (Figure 5), a **13** analogue, showed interesting antiviral activity (EC_50_ = 0.55 μM, MDCK cells infected with the flu A/HK/415742/2009 (H1N1) strain, PRA assay) coupled with a high selectivity index (CC_50_ = 125 μM, SI = 227). Compound **14** also inhibited viral replication of a panel of eight flu A strains in a dose-dependent manner with EC_50_’s ranging from 0.09 to 1.23 μM (SI from 101 to 1388). These results were confirmed by an *in vivo* assay using a mouse-adapted flu A/HK/415742/2009 (H1N1) strain, where mice intranasal treatment with **14** led to full mice protection. The mechanism of action of **14** was confirmed by a time-of-addition (TOA) assay, which showed that the compound decreased vRNA but not mRNA production at 3 h postinfection and decreased both mRNA and vRNA production at 6 h postinfection. A minireplicon assay confirmed its inhibitory effect on RdRP activity. Finally, an isothermal titration calorimetry (ITC) assay showed that **14** binds PA_C_ with a *K*_d_ value of 1.32 μM, while no binding was detected with PB1_N_. Molecular docking studies suggested that compound **14** might interact with a PA_C_ allosteric site rather than with a PB1 binding site, by forming H-bonds with residues D426, Q427, R582, and L585 (the original figure reporting the binding pose of compound **14** is reported in Figure S3). Thus, compound **14** could not be considered a real PA–PB1 inhibitor. Nevertheless, the conserved α-helix-8 region, where the allosteric binding site is located, plays a critical role in the interaction with PB1, and compound **14** binding might induce a conformational change in PA causing abrogation of the PA–PB1 interaction.

In 2017, Watanabe et al.^104^ screened *in silico* a library of 600,000 compounds to evaluate the binding energy of the ligands by using the crystal structure 2ZNL(74) as template. Among the 136 compounds selected as potential antiflu candidates, 99 were purchased and screened in a cell-based crystal violet (CV) assay, in which the virus infection-induced cytopathic effect in cells was observed. Data showed MIC values ≤ 20 μM for 14 compounds, which were evaluated in a surface plasmon resonance (SPR) analysis to determine their binding affinity with PA. Compound **15** (Figure 5) showed a good *K*_d_ value of 7.5 μM. Thus, it was investigated in a nuclear transportation-inhibition assay showing that the addition of the compound to PA– and PB1–*co*-transfected cells impaired intranuclear translocation of PA. It displayed an interesting antiflu activity in a PRA assay (EC_50_ = 0.47 μM, MDCK cells infected with the flu A/WSN/1933 (H1N1) strain) and in a CV assay (EC_50_ ranging from 0.53 to 0.92 μM, MDCK cells infected with a panel of four flu A and one flu B strains) without cytotoxicity up to 100 μM concentration (MDCK cells, CV assay). The ability of the compound to inhibit vRNA synthesis was also confirmed by Western blotting and TOA assays. Docking studies suggested that the binding site of compound **15** is located in the center of the PB1 binding site of PA (the original figure reporting the binding pose of compound **15** is reported in Figure S4).

In 2018, three independent groups reported on the identification of PA–PB1 disrupters by exploiting different approaches.

**Figure 6:**
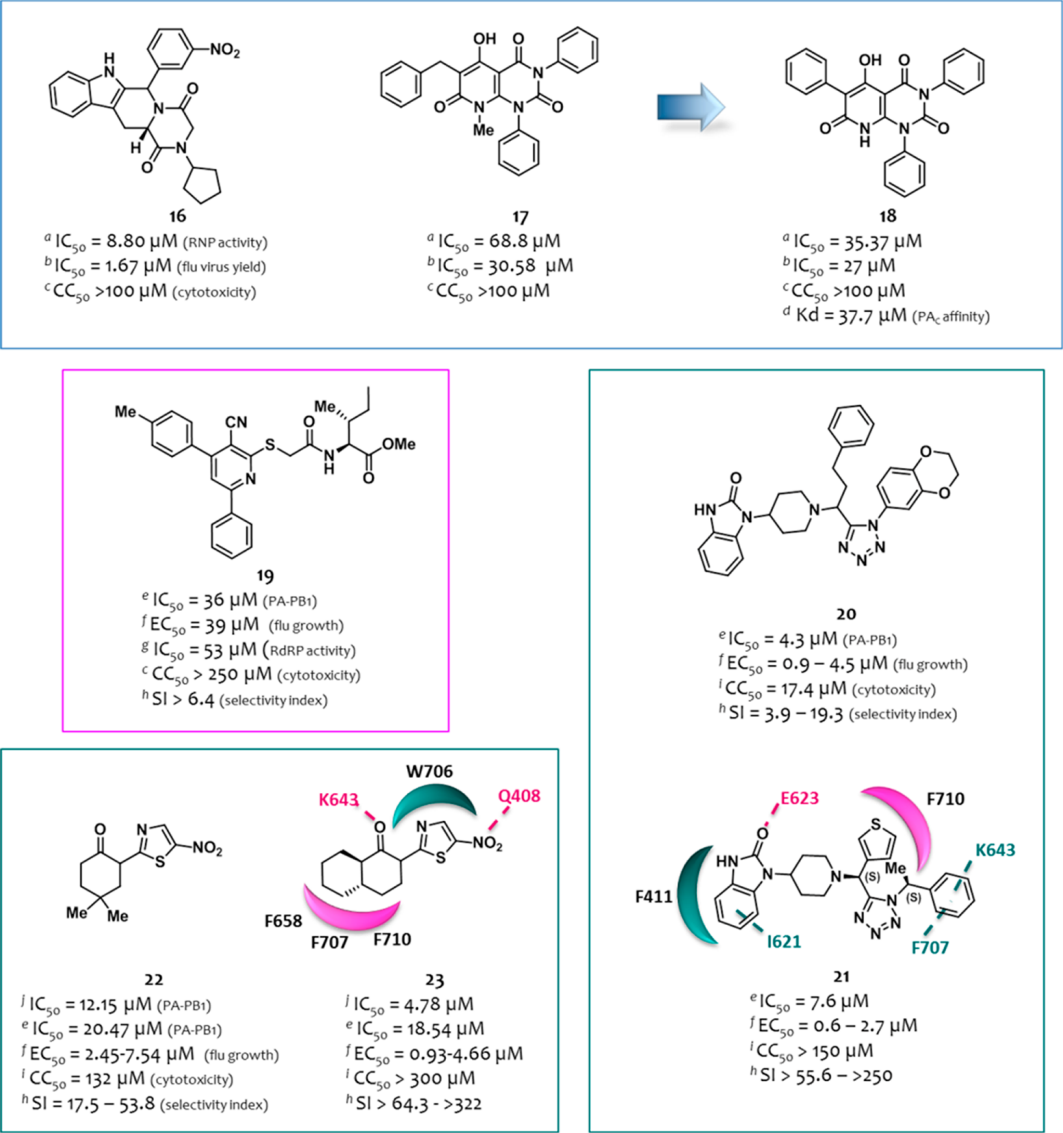
Structures and activities of the PA–PB1 interaction inhibitors identified by Lo et al.^105^ (in the blue box), D’Agostino et al.^106^ (in the magenta box), and Zhang et al.^108,109^ (in the teal boxes). For the definition of ^*e*^IC_50_, ^*f*^EC_50_, CC_50_, and *K*_d_, see the Figure 4 caption. ^*a*^IC_50_: compound concentration that reduces by 50% the RNP activity (RNP reconstitution reporter assay); ^*b*^IC_50_: compound concentration that inhibits 50% of viral yield in MDCK cells (viral yield assay); ^*c*^CC_50_, MTT assay (MDCK); ^*d*^*K*_d_, SPR assay; ^*e*^IC_50_, ELISA assay; ^*f*^EC_50_, PRA assay (MDCK cells); ^*g*^IC_50_, minireplicon assay; ^*h*^SI represents the ratio between CC_50_ and the highest/lowest EC_50_ values; ^*i*^CC_50_: NRU assay (MDCK cell growth); ^*j*^IC_50_, SLC assay. The figure is author created, while the original figures reporting the binding pose of compounds **19**, **21**, and **23** are reported in Figures S5–S7.

Lo et al.^105^ performed a SPR screening of an in-house library of 165 compounds against PA_C_ (residues 257–716), leading to the identification of two initial hit compounds, **16** and **17** (Figure 6). They attenuated the vRNP transcriptional activities (in a RNP reconstitution reporter assay) in a dose-dependent manner with IC_50_ values of 8.80 and 68.8 μM, respectively, and inhibited the flu A/WSN/1933 (H1N1) strain replication in a viral yield reduction assay (IC_50_ = 1.67 and 30.58 μM, respectively) without showing any cytotoxicity in both 293T and MDCK cells. Despite compound **16** showed the most promising antiviral profile, on the basis of the better solubility but above all the suitability to chemical manipulation, compound **17** has been selected to search for additional analogues. Thus, 13 commercially available analogues were purchased, and 10 derivatives were designed and synthesized. Among them, compound **18** (Figure 6) showed an improved ability to inhibit the vRNP activity (IC_50_ = 35.37 μM), maintaining the same profile of antiviral activity and cytotoxicity (IC_50_ = 27.0 μM and CC_50_ > 100 μM). Moreover, it showed dose-dependent inhibition of vRNP activity of four different flu A strains (flu A/WSN/1933 (H1N1), A/Japan/305/1957 (H2N2), A/HK/1/1968 (H3N2), and A/HK/156/1997 (H5N1) strains), even if the potency against H5N1 vRNP was weaker than those against the other strains. Additionally, for compound **18**, the ability to interact with the PA_C_ in both microscale thermophoresis (MST) and SPR assays was confirmed, with consistent *K*_d_ values at the micromolar level (38.2 and 37.7 μM, respectively). To gain information on the binding site of **18** within PA_C_, the authors investigated the discrepancy between the inhibition of H5N1 vRNP activity and that of other flu A strains. Sequence alignment of PA_C_ of all the tested strains revealed that H5N1 differs from the others in 17 residues, which are in close proximity to the Pol II interacting residues, viral promoter and vRNA binding region, and the PB1-binding cavity. Thus, the authors hypothesized that the binding site of compound **18** within PA_C_ could involve the above-mentioned binding sites.

A series of hybrid compounds was synthesized by D’Agostino et al.^106^ to further investigate the previously reported 3-cyano-4,6-diphenylpyridine class of PA–PB1 inhibitors,^99,107^ of which compound **6** (Figure 4) inhibited both the PA–PB1 interaction (IC_50_ = 35 μM, ELISA assay) and flu replication (EC_50_ = 9.2 μM, PRA assay, A/Puerto Rico/8/1934 (H1N1) strain) at nontoxic concentrations (CC_50_ > 250 μM).^99^ With the aim to increase the affinity of the 3-cyano-4,6-diphenylpyridine scaffold toward PA_C_ and thus enhance its ability to displace PB1_N_, the cyano-diphenylpyridine core was combined with the last three amino acids (M1-D2-V3) of the PB1_N_ peptide. In particular, tripeptidic (M–D–V), dipeptidic (D–V), and monoamino acid (V) methyl ester side chains were inserted at the C-2 position of the nucleus. Other amino acids (L, I, H, R, F, and G) were also exploited by synthesizing monoamino acid methyl ester derivatives. The best biological profile was shown by the mono acidic isoleucine derivative **19** (Figure 6), showing values of IC_50_ = 36 μM (PA–PB1), EC_50_ = 39 μM (flu A/Puerto Rico/8/1934 (H1N1)), and CC_50_ > 250 μM. It inhibited polymerase activity with an IC_50_ of 53 μM. For compound **19**, molecular docking and dynamic studies suggested a binding mode comparable to 3-cyano-4,6-diphenylpyridine previously reported by the authors such as compound **6** (Figure 1) (the original figure reporting the binding pose of compound **19** is reported in Figure S5).

With the aim to exploit a fast-track drug discovery approach for the identification of PA–PB1 inhibitors, Zhang et al.^108^ performed an *in silico* screening of 2000 compounds belonging to an in-house library of multicomponent reaction products by using the crystal structure 3CM8(72) as template. The selected top hits were then tested for the ability to inhibit the PA–PB1 subunit interaction by ELISA. Among them, compound **20** (Figure 6) showed PA–PB1 inhibition in a dose-dependent manner with an IC_50_ of 4.3 μM. This activity well translated in a good antiviral activity (EC_50_ from 0.9 to 4.5 μM) against five flu A(H1N1) and two flu B strains including oseltamivir-sensitive and oseltamivir-resistant strains (PRA assay, MDCK cells) at subcytotoxic concentrations (CC_50_ = 17.4 μM, neutral red uptake (NRU) assay, MDCK cells) with SI values from 3.9 to 19.3. Starting from compound **20**, a successive SAR study was then accomplished by exploring different moieties of the molecule and entailing the synthesis of a focused library of 23 derivatives prepared by the one-pot Ugi-azide four component reaction, also employing chiral starting materials. Among them, the (*S*,*S*) diastereoisomer derivative **21** (Figure 6) emerged as the most active. Compared to the parental compound **20**, it exhibited similar PA–PB1 inhibitory activity (IC_50_ = 7.6 μM) but improved antiviral activity not only against the flu A/WSN/1933 (H1N1) strain (EC_50_ = 0.7 μM) but also against a panel of human clinical isolates of six flu A and five flu B strains (EC_50_ from 0.6 to 2.7 μM, PRA assay). Moreover, compound **21** showed an inferior cytotoxicity in both MDCK and A549 cells (NRU assay) with CC_50_ values of 150 and of 98.1 μM, respectively (SI values from 55.6 to 250 in MDCK cells and from 36.3 to 163.5 in A549 cells). Successive mechanistic studies confirmed the inhibition of the PA–PB1 interaction as its antiviral mechanism of action. Indeed, the TOA experiment showed that the pretreatment of cells with the compound has little to no effect on viral replication, while it inhibited the intermediate stage of viral replication postviral fusion. Moreover, RT-qPCR experiments confirmed that it was able to inhibit vRNA, cRNA, and mRNA expression in a dose-dependent manner. Molecular docking studies suggested that compound **21** can be accommodated in the PB1-binding pocket of PA, forming extensive hydrophobic and multiple π–π interactions mainly through the phenyl ring with F707 and K643, the thiophene ring with F710, and the benzoimidazol-2-one phenyl ring with F411 and I621. In addition, the benzoimidazol-2-one carbonyl group is involved in a H-bond with E623 backbone amide NH (the original figure reporting the binding pose of compound **21** is reported in Figure S6). Finally, compound **21** was demonstrated to possess a higher *in vitro* genetic barrier to drug resistance than oseltamivir, since for up to 10 passages flu A/WSN/1933 (H1N1) remained sensitive to the compound when assayed in PRA (for oseltamivir carboxylate, the EC_50_ increased 10-fold at passage six and onward).

In late 2020, Zhang et al.^109^ published a further paper on the identification of PA–PB1 inhibitors by using an HTS approach. In particular, the authors developed an *in vitro* split luciferase complementation-based (SLC) assay for HTS, which was used to screen 10,000 compounds from the MyriaScreen Diversity Collection. Among them, 105 compounds displaying >95% inhibition at 20 μM concentration were tested for the antiviral activity in a virus-induced CPE assay. Compounds **22** and **23** (Figure 6) showed potent antiviral activity (MDCK cells infected with the flu A/WSN/1933 (H1N1) strain) at 10 μM. Both the compounds were able to interfere with the PA–PB1 heterodimerization in a dose-dependent manner in both the SLC assay (IC_50_ values of 12.15 and 4.78 μM, respectively) and ELISA assay (IC_50_ values of 20.47 and 18.54 μM, respectively). They also showed antiflu activity against seven flu A(H1N1), one flu A(H3N2), and two flu B strains including multiple drug-resistant strains (PRA assay, MDCK or AX-4 cells), with EC_50_ values ranging from 2.45 to 7.54 μM and from 0.93 to 4.66, respectively, at subcytotoxic concentrations (CC_50_ = 132 and >300 μM, respectively, NRU assay, MDCK cells). On the basis of the higher SI values (from >64.3 to >322), compound **23** was selected for further studies, showing potent inhibition against flu A(H1N1) and flu A(H3N2) strains at both low and high multiplicity of infections. Moreover, it reduced PA nuclear localization in PA–PB1 coexpressing cells highlighting the PA–PB1 interaction inhibition in a cellular context, and TOA studies suggested that **23** acts in the early phases of viral replication, analogously to baloxavir marboxil. Finally, it was able to reduce in a dose-dependent manner vRNA, cRNA, and mRNA levels, as shown by RT-qPCR as well as the NP and M1 protein expression levels, as measured by Western blot and immunofluorescence assays. Molecular docking studies performed for **23** within the PA_C_ cavity suggested several key interactions, i.e., a π–π interaction between the thiazole ring and W706, a hydrophobic interaction of the decalin ring with the pocket defined by aa F658, F707, and F710, and two H-bonds between the keto and nitro groups with K643 and Q408, respectively (the original figure reporting the binding pose of compound **23** is reported in Figure S7).

**Figure 7:**
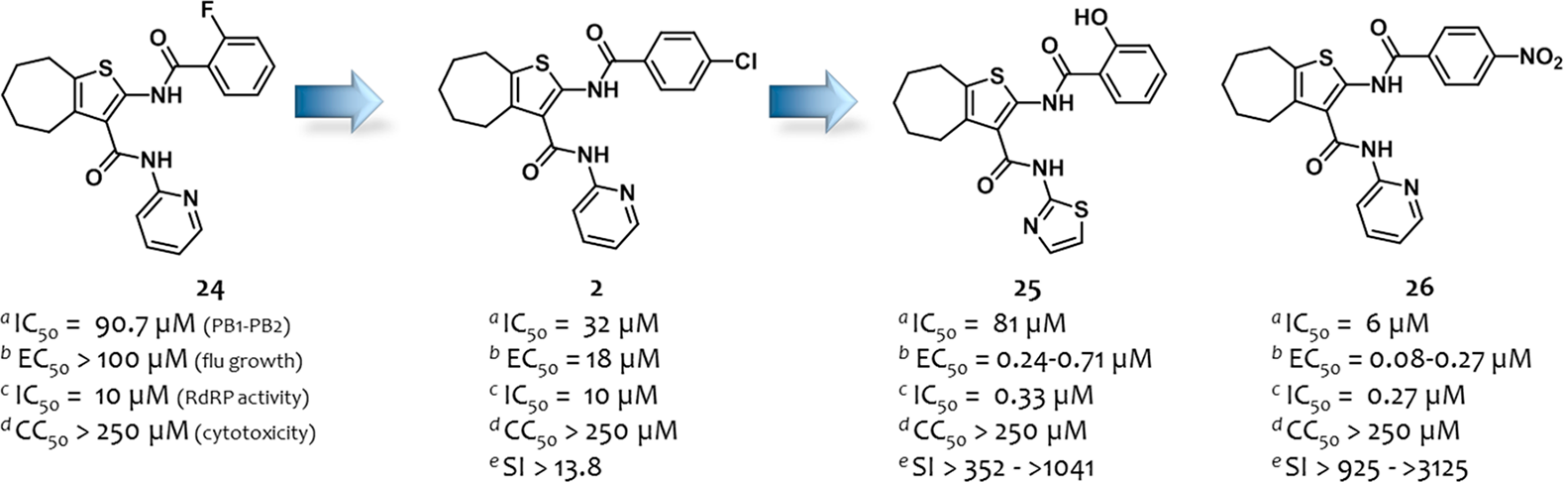
Structures and activities of PA–PB1 inhibitors identified by us.^111,112^ For the definition of IC_50_, EC_50_, and CC_50_, see the Figure 4 caption. ^*a*^IC_50_, ELISA assay; ^*b*^EC_50_, PRA assay (MDCK cells); ^*c*^IC_50_, minireplicon assay; ^*d*^CC_50_, MTT assay (MDCK cells); ^*e*^SI represents the ratio between CC_50_ and the highest/lowest EC_50_ values.

Our group has been working for years on the development of small molecule PA–PB1 complex formation inhibitors.^95,96,98,110−112^ The study started with a SBDD^95^ by screening 3 million small molecules from the ZINC database on PA_C_ from the crystal structures 3CM8.^72^ Among the 32 virtual hits identified, five showed the ability to inhibit the PA–PB1 interaction in an ELISA assay. The cyclohepthathiphene–3-carboxamide compound **24** (Figure 7) was subjected to a first optimization phase that led to the identification of compound **2**, which showed an improved ability to displace the PA–PB1 complex (IC_50_ = 32 μM) and, above all, acquired antiflu activity (EC_50_ = 18 μM) at nontoxic concentrations (CC_50_ > 250 μM).^96^ In 2017, a second optimization phase of cycloheptathiophene–3-carboxamide-based compounds was performed,^111^ exploring extensively both the aromatic rings at the C-2 and C-3 positions of the core. Six compounds with a selectivity index of >25 were identified with derivatives **25** and **26** (Figure 7), which emerged as the most active. Both compounds showed an improved antiflu activity (EC_50_ = 0.18 and 0.26 μM, respectively, flu A/Puerto Rico/8/1934 (H1N1) strain, MDCK cells) in a PRA assay but a weaker ability to interfere with PA–PB1 complex formation (IC_50_ = 69 and 65 μM, respectively, ELISA assay). However, derivative **26** showed a higher ability to inhibit the PA–PB1 interaction with an IC_50_ = 6.0 μM in an ELISA assay in which serum-free DMEM was used as medium instead of PBS.^112^ On the other hand, the weak anti-PA–PB1 activity of **25** was confirmed (IC_50_ = 81 μM). Nevertheless, both compounds **25** and **26** were potent inhibitors of flu RdRP activity in a minireplicon assay (IC_50_ = 0.33 and 0.27 μM, respectively), suggesting a different mechanism of polymerase inhibition for compound **25** than the interference with the PA–PB1 interaction. Both compounds **25** and **26** showed potent antiflu activity against a panel of five flu A and three flu B strains (EC_50_ values ranging from 0.24 to 0.71 μM and from 0.08 to 0.27 μM, respectively, PRA assay) and were potent inhibitors also in a virus yield reduction assay (EC_50_ = 0.41 and 2.9 μM, respectively). The propensity of cyclohepthathiophene–3-carboxamide-based derivatives to induce drug resistance was also evaluated by selecting *in vitro* flu strains under compound selective pressure. Of note, the activity of the compounds remained unvaried over the whole selection process (until 20/30 passages), suggesting that they are not prone to develop drug resistance *in vitro*.^112^

### PB1–PB2 Inhibitors

**Figure 8:**
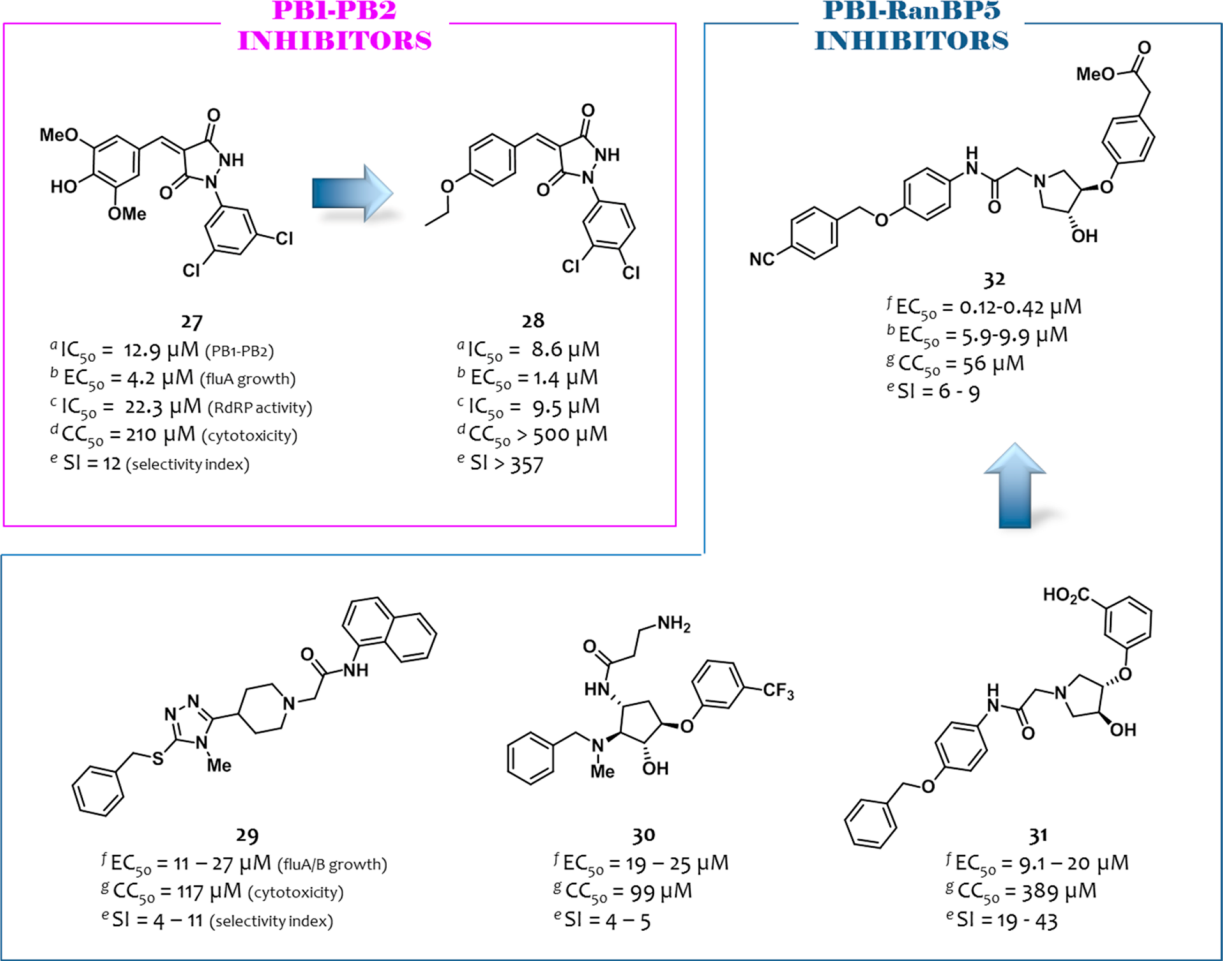
Structures and activities of PB1–PB2 inhibitors identified by Yuan et al.^77^ and PB1–RanBp5 inhibitors reported by Mohl et al.^71^ For the definition of ^*e*^IC_50_, ^*f*^EC_50_, CC_50_, SI, and *K*_d_, see the Figure 4 caption. ^*a*^IC_50_, ELISA assay; ^*b*^EC_50_: PRA assay (MDCK cells); ^*c*^IC_50_, minireplicon assay; ^*d*^CC_50_, MTT assay (MDCK cells); ^*e*^SI represents the ratio between CC_50_ and the highest/lowest EC_50_ values; ^*f*^EC_50_: IF assay (MDCK cells); ^*g*^CC_50_, CV-CTX assay (MDCK cells). The figure is author created, while the original figure reporting the binding pose of an analogue of compound **32** is reported in Figure S8.

In an effort to demonstrate the possibility of suppressing viral replication by abrogating the PB1–PB2 binding, in 2017, Yuan et al. initially evaluated the antiviral activity of a PB2_N_ derived peptide^113^ fused to Tat protein.^77^ Then, the authors set up a modified ELISA assay to screen PB1–PB2 inhibitors using full-length PB1 protein and biotinylated PB2_N_ peptide and screened a library of 950 compounds. Among them, compound **27** (Figure 8) showed an IC_50_ = 12.9 μM and was the sole to show dose-dependent antiflu activity (EC_50_ = 4.2 μM, MDCK cells infected with the flu A/HK/415742/2009 (H1N1) strain, PRA assay). On the basis of its chemical structure, 12 analogues with drug-like properties were purchased, of which the pyrazolidine-3,5-dione derivative **28** (Figure 8) was endowed with both anti-PB1-PB2 and antiviral activities (IC_50_ = 8.6 μM and EC_50_ = 1.4 μM). It also showed lower cytotoxicity (CC_50_ > 500 μM) than **27** (CC_50_ > 210 μM). When compound **28** was tested against a panel of six flu A strains, a strain/subtype-dependent inhibition of flu replication was observed. In particular, compound **28** treatment led to dose-dependent inhibition of the replication of flu A strains H1N1/pdm09, H7N9, and H9N2, but it was unable to inhibit H5N1 and H7N7 replication at the higher concentration used of 40 μM. For **28**, docking studies suggested a binding mode with a PB1_C_ domain similar to that of the PB2_N_ peptide. Nevertheless, all eight PB1 residues involved in binding with compound **28** were conserved among the six flu A strains, as shown by aligning sequences of their PB1_C_ residues (678–757).

### PB1–RanBP5 Inhibitors

As reported above, importin-β RanBP5 is a nuclear import factor that associates with PA–PB1 heterodimer and imports it into the nucleus. Therefore, analogously to PA–PB1 inhibitors, compounds able to block PA–PB1–RanBP5 binding might hinder RdRP heterotrimerization, thus inhibiting its functions.

In 2019, Mohl et al. reported a study focused on the identification of inhibitors targeting the interaction of PB1 with RanBP5.^71^ By using the flu A polymerase structure 4WSB,^31^ the Asinex PPI inhibitor library was virtually screened by docking the compounds to the predicted RanBP5 binding site of PB1, localized near the PB1 NLS containing domain. Eight compounds were selected that were used for library expression. In particular, a structure search for commercially available analogues followed by docking filtration led to the identification of five virtual hits, which were evaluated for the antiflu activity (three flu A strains and one flu B strain) in an immunofluorescence (IF) assay (MDCK cells) and for their cytotoxicity in a CV assay (MDCK cells). Among them, compounds **29**–**31** (Figure 8) showed EC_50_ values against the flu A/WSN/1933 (H1N1) strain of 15, 19, and 9.5 μM and CC_50_ values of 117, 99, and 389 μM, respectively. On the basis of the cost and feasibility of the chemical synthesis, compound **31** was selected for optimization, leading to the synthesis of 24 analogues. Structural modifications led to the identification of five compounds that showed a potent activity in the IF assay but resulted in less efficiency in the crystal violet cytopathic (CV-CPE) assay, also showing an increased cytotoxicity than **31**. The same behavior was shown by the compounds when evaluated against five flu A and B strains. The most balanced profile was demonstrated by compound **32** (EC_50_ = 0.12–0.42 μM in the IF assay, 3.7–44 μM in the CV-CPE assay, and CC_50_ = 56 μM in the CV-CTX assay), for which the antiflu activity was confirmed in an XTT-CPE and plaque assay (EC_50_ values of 5.9 and 9.9 μM, respectively). Docking studies were performed for a strict analogue of compound **32** within the RanBP5 binding site of PB1, suggesting key interactions with residues Y689, D685, and R680 of the anchor helix of PB1, which is important for the RanBP5 interaction (the original figure reporting the binding pose of the analogue of compound **32** is reported in Figure S8). Compound **32** was also evaluated for its resistance profile, showing no resistance development after ten passages in the IF assay. Moreover, it showed the ability to inhibit RdRP in a luciferase minigenome reporter assay in Vero and HepG2-hNTCP cells, while no activity was observed at a subcytotoxic concentration in 293T, MT-4, and Huh7 cell lines. Finally, the effect of compound **32** on PB1 and NP localization was studied in MDCK cells, showing that, analogously to PA–PB1 inhibitors, it prevents nuclear accumulation of PB1 and, consequently, NP export. Thus, although the inhibition of the PA–PB1–RanBP5 interaction was not demonstrated by a PPI inhibition assay and the compounds suffered from a certain cytotoxicity, this study confirmed the validity of targeting this viral–host interaction to achieve the antiflu activity by exploiting an alternative mechanism.

## Concluding Remarks and Future Perspectives

Flu infections are responsible for annual epidemics associated with high medical costs. Flu viruses also generate unpredictable pandemic outbreaks; indeed, the WHO included global influenza pandemic infections in the list of “10 Threats to Global Health”.^114^

Unfortunately, the antiviral armamentarium is limited to a few licensed drugs for which the development of drug resistance limits their range of use. However, the most recently approved compounds or those in the pipeline act by inhibiting each of the three subunits of RdRP, which therefore is emerging as a privileged drug target.

An innovative approach to interfere with the RdRp functions entails its inhibition through dissociative compounds. For an efficient and successful transcription and replication in host cells, RdRP subunits must interact among each other and rely heavily on the association with host proteins, which are essential cofactors for promoting these steps. Thus, numerous PPIs by RdRP occur during its journey within the viral replicative cycle, which could be exploited for the discovery of alternative antiflu drugs.

Here, we have summarized all the small molecules recently reported to be endowed with antiflu activity thanks to the inhibition of one of such PPIs. Among them, the PA–PB1 interface was the most explored with intense efforts made in recent years that permitted one to enlarge the range of active chemotypes as well as reach very potent antiflu activity. Triazolopyrimidinol derivative **14** and cycloheptathiophene–3-carboxamide derivative **26** were among the most active with EC_50_ values against a panel of flu strains ranging from 0.09 to 1.23 μM (eight flu A strains) and 0.08 to 0.27 μM (five flu A and three flu B strains), respectively. Compound **26** resulted in the most selectivity with SI values >3000, and its activity was comparable to or even higher than that observed for polymerase inhibitor favipiravir against flu A and B strains (EC_50_ values ranging from 0.083 to 3.1 μM, SI > 2000).^115^ The propensity of PA–PB1 inhibitors to induce drug resistance was also evaluated, showing that they are impressively refractory to select drug-resistant viral variants under high-dose selective pressure. This is the case of PA–PB1 inhibitors **21** (10 passages) and **26** (20/30 passages). Notably, this behavior was also shown by compound **32** (10 passages), which inhibited another PPI, such as PB1–RanBP5.

The results obtained for PA–PB1 inhibitors strengthened the validity of PPIs by RdRP as drug targets to obtain antiflu compounds. Such inhibitors could have great advantages over the inhibitors of the single RdRP subunits. First of all, a broad spectrum antiflu activity is expected by PPI inhibitors, since residues of RdRP subunits involved in the binding are highly conserved among different flu strains. It is worth mentioning that the range of antiviral activity of the PB2 inhibitor pimodivir is limited to flu A, as this compound was found to be ineffective against flu B, while the PA inhibitor baloxavir marboxil is able to also inhibit flu B strains, but at concentrations 10-fold higher than those observed against flu A strains.^35,49^

Second, PPI inhibitors should be less prone to develop drug–resistant viruses, since to escape from the drug pressure of PPI inhibitors, flu viruses should acquire simultaneous mutations in the binding portion of both viral subunits in order to restore an efficient RdRP complex assembly without a substantial loss of the viral fitness. On the other hand, resistant mutations to both pimodivir and baloxavir marboxil have been selected *in vitro* or in treated patients.^48,49^

However, further work remains to be done to make inhibitors of PPIs by RdRP real candidates. In particular: (i) only computational studies were done to study their binding mode, while no cocrystallization studies and mutagenesis experiments were performed, which could facilitate the optimization of the compounds; (ii) coadministration with agents acting through different mechanisms of action remains to be determined; (iii) the behavior of the best compounds in an animal model was not evaluated, with the only exception of compound **14**, for which the *in vivo* activity was measured, showing encouraging results. Additionally, the solubility of PA–PB1 inhibitors should be improved. Indeed, by interacting with interfaces generally flat and highly hydrophobic, PPI inhibitors often suffer from low solubility; in agreement, the PA_C_ cavity is characterized by hydrophobic pockets, and the efficient binding by small molecules is mainly ensured by hydrophobic interactions. Only a few of the molecules herein reported were identified in the search for aqueous soluble compounds, but the pharmacokinetic properties should be considered also in the hit-to-lead optimization phase, which was aimed at improving the affinity of the molecules with PA_C_ by increasing their hydrophobicity at the expense of solubility.

From our survey, it clearly emerged that PPIs other than PA–PB1 should be worth exploiting. Regarding viral–viral interactions, the structure of the RdRp dimer has been recently reported and is still unexplored, while only one research group has focused on the PB1–PB2 interaction although the crystal structure was reported in 2009. Nevertheless, as stated by Yuan et al. who explored both PA–PB1 and PB1–PB2 interfaces, PA–PB1 appeared as a preferred drug target for searching flu RdRP complex formation inhibitors.^116^

Concerning RdRP-host PPIs, different interfaces can be exploited. The interference with host cell factors involved in viral replication rather than viral components may open new perspectives to counteract flu infection while reducing the development of drug resistance. One of the most promising PPIs occurring between RdRP subunits and host factors could be PA–Pol II CTD, but no inhibitors were reported to date. One class of PB1–RanBP5 inhibitors with antiflu activity has been recently reported, demonstrating that interfering with nuclear localization of the RdRP subunits can be feasible for achieving novel antiflu compounds. Further studies are instead required to determine if the interaction between PB2 and host factors importin-α and ANP32 can be a good drug target, due to the presence of residues involved in host adaptation in the proximity of the binding sequence.

Overall, the insights outlined in this Review clearly suggested that PPI inhibition, although at its infancy, is a highly promising strategy to inhibit flu RdRP functions and thus to identify innovative antiflu compounds characterized by numerous advantages over classic RdRP inhibitors. Moreover, the Review collects for the first time all the interactions occurring by RdRP subunits that could be disrupted by small molecules. Their benefits and drawbacks as a drug target were analyzed, and this work offers scientists involved in the antiflu research field the opportunity to apply the PPI inhibition strategy toward new interfaces still unexplored.

## Supporting Information Available

Original figures reporting the binding pose of compounds **1**–**10**, **14**, **15**, **19**, **21**, and **23** and of an analogue of compound **32** (PDF)

## Abbreviations

ANP32: acidic nuclear phosphoproteins 32
CTD: carboxy-terminal domain
cRNP: complementary ribonucleoprotein
cRNA: complementary RNA
CV: crystal violet
CV-CPE: crystal violet cytopathic effect
CPE: cytopathic effect
CV-CTX: crystal violet cytotoxicity
ELISA: enzyme-linked immunosorbent assay
Hsp90: heat shock protein 90
HA: hemagglutinin
HTS: high throughput screening
HPAI: highly pathogenic avian influenza
IF: immunofluorescence
flu: influenza
ITC: isothermal titration calorimetry
LPAI: low pathogenicity avian influenza
M1: matrix protein 1
M2: membrane matrix protein
MST: microscale thermophoresis
NA: neuraminidase
NRU: neutral red uptake
NLS: nuclear localization signal
NP: nucleoprotein
PRA: plaque reduction assay
PA: polymerase acidic protein
PB1: polymerase basic protein 1
PB2: polymerase basic protein 2
PPI: protein–protein interaction
RanBP5: Ran-binding protein 5
Pol II: RNA polymerase II
RdRP: RNA-dependent RNA polymerase
SLC: split luciferase complementation-based
SAR: structure–activity relationship
SBDD: structure-based drug design
TOA: time-of-addition assay
vRNP: viral ribonucleoprotein
vRNA: viral RNA

## References

[ref1] Taubenberger J. K.; Kash J. C.; Morens D. M. (2019) The 1918 Influenza Pandemic: 100 Years of Questions Answered and Unanswered. Sci. Transl. Med. 11, eaau5485 10.1126/scitranslmed.aau5485. 31341062 PMC11000447

[ref2] Bouvier N. M.; Palese P. (2008) The Biology of Influenza Viruses. Vaccine 26 (SUPPL. 4), D49 10.1016/j.vaccine.2008.07.039. 19230160 PMC3074182

[ref3] Garten R. J.; Davis C. T.; Russell C. A.; Shu B.; Lindstrom S.; Balish A.; Sessions W. M.; Xu X.; Skepner E.; Deyde V.; Okomo-Adhiambo M.; Gubareva L.; Barnes J.; Smith C. B.; Emery S. L.; Hillman M. J.; Rivailler P.; Smagala J.; De Graaf M.; Burke D. F.; Fouchier R. A. M.; Pappas C.; Alpuche-Aranda C. M.; López-Gatell H.; Olivera H.; López I.; Myers C. A.; Faix D.; Blair P. J.; Yu C.; Keene K. M.; Dotson P. D.; Boxrud D.; Sambol A. R.; Abid S. H.; St. George K.; Bannerman T.; Moore A. L.; Stringer D. J.; Blevins P.; Demmler-Harrison G. J.; Ginsberg M.; Kriner P.; Waterman S.; Smole S.; Guevara H. F.; Belongia E. A.; Clark P. A.; Beatrice S. T.; Donis R.; Katz J.; Finelli L.; Bridges C. B.; Shaw M.; Jernigan D. B.; Uyeki T. M.; Smith D. J.; Klimov A. I.; Cox N. J. (2009) Antigenic and Genetic Characteristics of Swine-Origin 2009 A(H1N1) Influenza Viruses Circulating in Humans. Science 325, 197–201. 10.1126/science.1176225. 19465683 PMC3250984

[ref4] Dowdle W. R. (1999) Influenza A Virus Recycling Revisited. Bull. World Heal. Organ. 77, 820–828. PMC2557748 10593030

[ref5] World Health Organization (accessed 2020-09-04) Influenza, http://www.who.int/influenza.

[ref6] Lu Y.; Landreth S.; Gaba A.; Hlasny M.; Liu G.; Huang Y.; Zhou Y. (2019) In Vivo Characterization of Avian Influenza a (H5N1) and (H7N9) Viruses Isolated from Canadian Travelers. Viruses 11, 193–205. 10.3390/v11020193. PMC6409709 30813415

[ref7] Peng Y.; Li X.; Zhou H.; Wu A.; Dong L.; Zhang Y.; Gao R.; Bo H.; Yang L.; Wang D.; Lin X.; Jin M.; Shu Y.; Jiang T. (2017) Continual Antigenic Diversification in China Leads to Global Antigenic Complexity of Avian Influenza H5N1 Viruses. Sci. Rep. 7, 43566–43577. 10.1038/srep43566. 28262734 PMC5337931

[ref8] Wang X.; Jiang H.; Wu P.; Uyeki T. M.; Feng L.; Lai S.; Wang L.; Huo X.; Xu K.; Chen E.; Wang X.; He J.; Kang M.; Zhang R.; Zhang J.; Wu J.; Hu S.; Zhang H.; Liu X.; Fu W.; Ou J.; Wu S.; Qin Y.; Zhang Z.; Shi Y.; Zhang J.; Artois J.; Fang V. J.; Zhu H.; Guan Y.; Gilbert M.; Horby P. W.; Leung G. M.; Gao G. F.; Cowling B. J.; Yu H. (2017) Epidemiology of Avian Influenza A H7N9 Virus in Human Beings across Five Epidemics in Mainland China, 2013–17: An Epidemiological Study of Laboratory-Confirmed Case Series. Lancet Infect. Dis. 17, 822–832. 10.1016/S1473-3099(17)30323-7. 28583578 PMC5988584

[ref9] Houser K.; Subbarao K. (2015) Influenza Vaccines: Challenges and Solutions. Cell Host Microbe 17, 295–300. 10.1016/j.chom.2015.02.012. 25766291 PMC4362519

[ref10] Krammer F.; Palese P. (2015) Advances in the Development of Influenza Virus Vaccines. Nat. Rev. Drug Discovery 14, 167–182. 10.1038/nrd4529. 25722244

[ref11] Principi N.; Camilloni B.; Alunno A.; Polinori I.; Argentiero A.; Esposito S. (2019) Drugs for Influenza Treatment: Is There Significant News?. Front. Med. 6, 109–115. 10.3389/fmed.2019.00109. PMC6546914 31192211

[ref12] Gubareva L. V.; Kaiser L.; Hayden F. G. (2000) Influenza Virus Neuraminidase Inhibitors. Lancet 355, 827–835. 10.1016/S0140-6736(99)11433-8. 10711940

[ref13] Fiore A. E.; Fry A.; Shay D.; et al. (2011) Antiviral Agents for the Treatment and Chemoprophylaxis of Influenza - Recommendations of the Advisory Committee on Immunization Practices (ACIP). MMWR Recomm. Rep. 60, 1–24. 21248682

[ref14] Watanabe A.; Chang S. C.; Kim M. J.; Chu D. W. S.; Ohashi Y. (2010) Long-Acting Neuraminidase Inhibitor Laninamivir Octanoate versus Oseltamivir for Treatment of Influenza: A Double-Blind, Randomized, Noninferiority Clinical Trial. Clin. Infect. Dis. 51, 1167–1175. 10.1086/656802. 20936975

[ref15] Sugaya N.; Ohashi Y. (2010) Long-Acting Neuraminidase Inhibitor Laninamivir Octanoate (CS-8958) versus Oseltamivir as Treatment for Children with Influenza Virus Infection. Antimicrob. Agents Chemother. 54, 2575–2582. 10.1128/AAC.01755-09. 20368393 PMC2876358

[ref16] Barroso L.; Treanor J.; Gubareva L.; Hayden F. G. (2005) Efficacy and Tolerability of the Oral Neuraminidase Inhibitor Peramivir in Experimental Human Influenza: Randomized, Controlled Trials for Prophylaxis and Treatment. Antivir. Ther. 10, 901–910. 16430195

[ref17] Furuta Y.; Gowen B. B.; Takahashi K.; Shiraki K.; Smee D. F.; Barnard D. L. (2013) Favipiravir (T-705), a Novel Viral RNA Polymerase Inhibitor. Antiviral Res. 100, 446–454. 10.1016/j.antiviral.2013.09.015. 24084488 PMC3880838

[ref18] Jones J. C.; Marathe B. M.; Lerner C.; Kreis L.; Gasser R.; Pascua P. N. Q.; Najera I.; Govorkova E. A. (2016) A Novel Endonuclease Inhibitor Exhibits Broad-Spectrum Anti-Influenza Virus Activity In Vitro. Antimicrob. Agents Chemother. 60, 5504–5514. 10.1128/AAC.00888-16. 27381402 PMC4997863

[ref19] Toots M.; Plemper R. K. (2020) Next-Generation Direct-Acting Influenza Therapeutics. Transl. Res. 220, 33–42. 10.1016/j.trsl.2020.01.005. 32088166 PMC7102518

[ref20] Dunning J.; Baillie J. K.; Cao B.; Hayden F. G. (2014) Antiviral Combinations for Severe Influenza. Lancet Infect. Dis. 14, 1259–1270. 10.1016/S1473-3099(14)70821-7. 25213733 PMC7164787

[ref21] Mifsud E. J.; Hayden F. G.; Hurt A. C. (2019) Antivirals Targeting the Polymerase Complex of Influenza Viruses. Antiviral Res. 169, 104545–104554. 10.1016/j.antiviral.2019.104545. 31247246

[ref22] Zhang J.; Hu Y.; Musharrafieh R.; Yin H.; Wang J. (2019) Focusing on the Influenza Virus Polymerase Complex: Recent Progress in Drug Discovery and Assay Development. Curr. Med. Chem. 26, 2243–2263. 10.2174/0929867325666180706112940. 29984646 PMC6426683

[ref23] Giacchello I.; Musumeci F.; D’Agostino I.; Greco C.; Grossi G.; Schenone S. (2020) Insights into RNA-Dependent RNA Polymerase Inhibitors as Anti-Influenza Virus Agents. Curr. Med. Chem. 27, 1 10.2174/0929867327666200114115632. 31942843

[ref24] Wandzik J. M.; Kouba T.; Cusack S. (2020) Structure and Function of Influenza Polymerase. Cold Spring Harbor Perspect. Med. 9, 38372–38391. 10.1101/cshperspect.a038372. PMC8415296 32341065

[ref25] Pflug A.; Lukarska M.; Resa-Infante P.; Reich S.; Cusack S. (2017) Structural Insights into RNA Synthesis by the Influenza Virus Transcription-Replication Machine. Virus Res. 234, 103–117. 10.1016/j.virusres.2017.01.013. 28115197

[ref26] Fodor E.; te Velthuis A. J. W. (2020) Structure and Function of the Influenza Virus Transcription and Replication Machinery. Cold Spring Harbor Perspect. Med. 10, a038398 10.1101/cshperspect.a038398. PMC7334866 31871230

[ref27] Stevaert A.; Naesens L. (2016) The Influenza Virus Polymerase Complex: An Update on Its Structure, Functions, and Significance for Antiviral Drug Design. Med. Res. Rev. 36, 1127–1173. 10.1002/med.21401. 27569399 PMC5108440

[ref28] Wandzik J. M.; Kouba T.; Karuppasamy M.; Pflug A.; Drncova P.; Provaznik J.; Azevedo N.; Cusack S. (2020) A Structure-Based Model for the Complete Transcription Cycle of Influenza Polymerase. Cell 181, 877–893. 10.1016/j.cell.2020.03.061. 32304664

[ref29] Kouba T.; Drncová P.; Cusack S. (2019) Structural Snapshots of Actively Transcribing Influenza Polymerase. Nat. Struct. Mol. Biol. 26, 460–470. 10.1038/s41594-019-0232-z. 31160782 PMC7610713

[ref30] Pflug A.; Guilligay D.; Reich S.; Cusack S. (2014) Structure of Influenza A Polymerase Bound to the Viral RNA Promoter. Nature 516, 355–360. 10.1038/nature14008. 25409142

[ref31] Reich S.; Guilligay D.; Pflug A.; Malet H.; Berger I.; Crépin T.; Hart D.; Lunardi T.; Nanao M.; Ruigrok R. W. H.; Cusack S. (2014) Structural Insight into Cap-Snatching and RNA Synthesis by Influenza Polymerase. Nature 516, 361–366. 10.1038/nature14009. 25409151

[ref32] Hengrung N.; El Omari K.; Serna Martin I.; Vreede F. T.; Cusack S.; Rambo R. P.; Vonrhein C.; Bricogne G.; Stuart D. I.; Grimes J. M.; Fodor E. (2015) Crystal Structure of the RNA-Dependent RNA Polymerase from Influenza C Virus. Nature 527, 114–117. 10.1038/nature15525. 26503046 PMC4783868

[ref33] Fan H.; Walker A. P.; Carrique L.; Keown J. R.; Serna Martin I.; Karia D.; Sharps J.; Hengrung N.; Pardon E.; Steyaert J.; Grimes J. M.; Fodor E. (2019) Structures of Influenza A Virus RNA Polymerase Offer Insight into Viral Genome Replication. Nature 573, 287–290. 10.1038/s41586-019-1530-7. 31485076 PMC6795553

[ref34] Takashita E. (2020) Influenza Polymerase Inhibitors: Mechanisms of Action and Resistance. Cold Spring Harbor Perspect. Med. 10, a038687 10.1101/cshperspect.a038687. PMC8091960 32122918

[ref35] Clark M. P.; Ledeboer M. W.; Davies I.; Byrn R. A.; Jones S. M.; Perola E.; Tsai A.; Jacobs M.; Nti-Addae K.; Bandarage U. K.; Boyd M. J.; Bethiel R. S.; Court J. J.; Deng H.; Duffy J. P.; Dorsch W. A.; Farmer L. J.; Gao H.; Gu W.; Jackson K.; Jacobs D. H.; Kennedy J. M.; Ledford B.; Liang J.; Maltais F.; Murcko M.; Wang T.; Wannamaker M. W.; Bennett H. B.; Leeman J. R.; McNeil C.; Taylor W. P.; Memmott C.; Jiang M.; Rijnbrand R.; Bral C.; Germann U.; Nezami A.; Zhang Y.; Salituro F. G.; Bennani Y. L.; Charifson P. S. (2014) Discovery of a Novel, First-in-Class, Orally Bioavailable Azaindole Inhibitor (VX-787) of Influenza PB2. J. Med. Chem. 57, 6668–6678. 10.1021/jm5007275. 25019388

[ref36] Finberg R. W.; Lanno R.; Anderson D.; Fleischhackl R.; van Duijnhoven W.; Kauffman R. S.; Kosoglou T.; Vingerhoets J.; Leopold L. (2019) Phase 2b Study of Pimodivir (JNJ-63623872) as Monotherapy or in Combination With Oseltamivir for Treatment of Acute Uncomplicated Seasonal Influenza A: TOPAZ Trial. J. Infect. Dis. 219, 1026–1034. 10.1093/infdis/jiy547. 30428049

[ref37] Witkowski J. T.; Robins R. K.; Sidwell R. W.; Simon L. N. (1972) Design, Synthesis, and Broad Spectrum Antiviral Activity of 1-β-D-Ribofuranosyl-1,2,4-Triazole-3-Carboxamide and Related Nucleosides. J. Med. Chem. 15, 1150–1154. 10.1021/jm00281a014. 4347550

[ref38] Sidwell R. W.; Huffman J. H.; Khare G. P.; Allen L. B.; Witkowski J. T.; Robins R. K. (1972) Broad-Spectrum Antiviral Activity of Virazole: 1-β-D-Ribofuranosyl-1, 2,4-Triazole-3-Carboxamide. Science 177, 705–706. 10.1126/science.177.4050.705. 4340949

[ref39] Toots M.; Yoon J. J.; Cox R. M.; Hart M.; Sticher Z. M.; Makhsous N.; Plesker R.; Barrena A. H.; Reddy P. G.; Mitchell D. G.; Shean R. C.; Bluemling G. R.; Kolykhalov A. A.; Greninger A. L.; Natchus M. G.; Painter G. R.; Plemper R. K. (2019) Characterization of Orally Efficacious Influenza Drug with High Resistance Barrier in Ferrets and Human Airway Epithelia. Sci. Transl. Med. 11, eaax5866 10.1126/scitranslmed.aax5866. 31645453 PMC6848974

[ref40] Toots M.; Yoon J. J.; Hart M.; Natchus M. G.; Painter G. R.; Plemper R. K. (2020) Quantitative Efficacy Paradigms of the Influenza Clinical Drug Candidate EIDD-2801 in the Ferret Model. Transl. Res. 218, 16–28. 10.1016/j.trsl.2019.12.002. 31945316 PMC7568909

[ref41] Yogaratnam J.; Rito J.; Kakuda T. N.; Fennema H.; Gupta K.; Jekle C. A.; Mitchell T.; Boyce M.; Sahgal O.; Balaratnam G.; Chanda S.; Van Remoortere P.; Symons J. A.; Fry J. (2019) Antiviral Activity, Safety, and Pharmacokinetics of AL-794, a Novel Oral Influenza Endonuclease Inhibitor: Results of an Influenza Human Challenge Study. J. Infect. Dis. 219, 177–185. 10.1093/infdis/jiy410. 30053042

[ref42] Yuan S.; Chu H.; Singh K.; Zhao H.; Zhang K.; Kao R. Y. T.; Chow B. K. C.; Zhou J.; Zheng B. J. (2016) A Novel Small-Molecule Inhibitor of Influenza A Virus Acts by Suppressing PA Endonuclease Activity of the Viral Polymerase. Sci. Rep. 6, 22880–22892. 10.1038/srep22880. 26956222 PMC4783701

[ref43] Wilson S. Z.; Knight V.; Wyde P. R.; Drake S.; Couch R. B. (1980) Amantadine and Ribavirin Aerosol Treatment of Influenza A and B Infection in Mice. Antimicrob. Agents Chemother. 17, 642–648. 10.1128/AAC.17.4.642. 7396454 PMC283845

[ref44] Wyde P. R.; Wilson S. Z.; Gilbert B. E.; Smith R. H. A. (1986) Protection of Mice from Lethal Influenza Virus Infection with High Dose-Short Duration Ribavirin Aerosol. Antimicrob. Agents Chemother. 30, 942–944. 10.1128/AAC.30.6.942. 3813516 PMC180625

[ref45] Snell N. J. C. (2001) Ribavirin - Current Status of a Broad Spectrum Antiviral Agent. Expert Opin. Pharmacother. 2, 1317–1324. 10.1517/14656566.2.8.1317. 11585000

[ref46] Beigel J. H.; Nam H. H.; Adams P. L.; Krafft A.; Ince W. L.; El-Kamary S. S.; Sims A. C. (2019) Advances in Respiratory Virus Therapeutics – A Meeting Report from the 6th Isirv Antiviral Group Conference. Antiviral Res. 167, 45–67. 10.1016/j.antiviral.2019.04.006. 30974127 PMC7132446

[ref47] Pettersen E. F.; Goddard T. D.; Huang C. C.; Couch G. S.; Greenblatt D. M.; Meng E. C.; Ferrin T. E. (2004) UCSF Chimera - A Visualization System for Exploratory Research and Analysis. J. Comput. Chem. 25, 1605–1612. 10.1002/jcc.20084. 15264254

[ref48] Byrn R. A.; Jones S. M.; Bennett H. B.; Bral C.; Clark M. P.; Jacobs M. D.; Kwong A. D.; Ledeboer M. W.; Leeman J. R.; McNeil C. F.; Murcko M. A.; Nezami A.; Perola E.; Rijnbrand R.; Saxena K.; Tsai A. W.; Zhou Y.; Charifson P. S. (2015) Preclinical Activity of VX-787, a First-in-Class, Orally Bioavailable Inhibitor of the Influenza Virus Polymerase PB2 Subunit. Antimicrob. Agents Chemother. 59, 1569–1582. 10.1128/AAC.04623-14. 25547360 PMC4325764

[ref49] Noshi T.; Kitano M.; Taniguchi K.; Yamamoto A.; Omoto S.; Baba K.; Hashimoto T.; Ishida K.; Kushima Y.; Hattori K.; Kawai M.; Yoshida R.; Kobayashi M.; Yoshinaga T.; Sato A.; Okamatsu M.; Sakoda Y.; Kida H.; Shishido T.; Naito A. (2018) In Vitro Characterization of Baloxavir Acid, a First-in-Class Cap-Dependent Endonuclease Inhibitor of the Influenza Virus Polymerase PA Subunit. Antiviral Res. 160, 109–117. 10.1016/j.antiviral.2018.10.008. 30316915

[ref50] Pflug A.; Gaudon S.; Resa-Infante P.; Lethier M.; Reich S.; Schulze W. M.; Cusack S. (2018) Capped RNA Primer Binding to Influenza Polymerase and Implications for the Mechanism of Cap-Binding Inhibitors. Nucleic Acids Res. 46, 956–971. 10.1093/nar/gkx1210. 29202182 PMC5778463

[ref51] Delang L.; Abdelnabi R.; Neyts J. (2018) Favipiravir as a Potential Countermeasure against Neglected and Emerging RNA Viruses. Antiviral Res. 153, 85–94. 10.1016/j.antiviral.2018.03.003. 29524445

[ref52] Furuta Y.; Komeno T.; Nakamura T. (2017) Favipiravir (T-705), a Broad Spectrum Inhibitor of Viral RNA Polymerase. Proc. Jpn. Acad., Ser. B 93, 449–463. 10.2183/pjab.93.027. 28769016 PMC5713175

[ref53] Hayden F. G.; Shindo N. (2019) Influenza Virus Polymerase Inhibitors in Clinical Development. Curr. Opin. Infect. Dis. 32, 176–186. 10.1097/QCO.0000000000000532. 30724789 PMC6416007

[ref54] Peacock T. P.; Sheppard C. M.; Staller E.; Barclay W. S. (2019) Host Determinants of Influenza RNA Synthesis. Annu. Rev. Virol. 6, 215–233. 10.1146/annurev-virology-092917-043339. 31283439

[ref55] Zhao M.; Wang L.; Li S. (2017) Influenza A Virus-Host Protein Interactions Control Viral Pathogenesis. Int. J. Mol. Sci. 18, 1673–1688. 10.3390/ijms18081673. PMC5578063 28763020

[ref56] Deng T.; Engelhardt O. G.; Thomas B.; Akoulitchev A. V.; Brownlee G. G.; Fodor E. (2006) Role of Ran Binding Protein 5 in Nuclear Import and Assembly of the Influenza Virus RNA Polymerase Complex. J. Virol. 80, 11911–11919. 10.1128/JVI.01565-06. 17005651 PMC1676300

[ref57] Hutchinson E. C.; Orr O. E.; Liu S. M.; Engelhardt O. G.; Fodor E. (2011) Characterization of the Interaction between the Influenza A Virus Polymerase Subunit PB1 and the Host Nuclear Import Factor Ran-Binding Protein 5. J. Gen. Virol. 92, 1859–1869. 10.1099/vir.0.032813-0. 21562121

[ref58] Boivin S.; Hart D. J. (2011) Interaction of the Influenza A Virus Polymerase PB2 C-Terminal Region with Importin α Isoforms Provides Insights into Host Adaptation and Polymerase Assembly. J. Biol. Chem. 286, 10439–10448. 10.1074/jbc.M110.182964. 21216958 PMC3060497

[ref59] Resa-Infante P.; Jorba N.; Zamarreño N.; Fernández Y.; Juárez S.; Ortín J. (2008) The Host-Dependent Interaction of α-Importins with Influenza PB2 Polymerase Subunit Is Required for Virus RNA Replication. PLoS One 3, e3904 10.1371/journal.pone.0003904. 19066626 PMC2588535

[ref60] Tarendeau F.; Boudet J.; Guilligay D.; Mas P. J.; Bougault C. M.; Boulo S.; Baudin F.; Ruigrok R. W. H.; Daigle N.; Ellenberg J.; Cusack S.; Simorre J. P.; Hart D. J. (2007) Structure and Nuclear Import Function of the C-Terminal Domain of Influenza Virus Polymerase PB2 Subunit. Nat. Struct. Mol. Biol. 14, 229–233. 10.1038/nsmb1212. 17310249

[ref61] Pumroy R. A.; Ke S.; Hart D. J.; Zachariae U.; Cingolani G. (2015) Molecular Determinants for Nuclear Import of Influenza A PB2 by Importin α Isoforms 3 and 7. Structure 23, 374–384. 10.1016/j.str.2014.11.015. 25599645 PMC4346194

[ref62] Chin A. W. H.; Leong N. K. C.; Nicholls J. M.; Poon L. L. M. (2017) Characterization of Influenza A Viruses with Polymorphism in PB2 Residues 701 and 702. Sci. Rep. 7, 1–12. 10.1038/s41598-017-11625-y. 28900145 PMC5595998

[ref63] Li Z.; Chen H.; Jiao P.; Deng G.; Tian G.; Li Y.; Hoffmann E.; Webster R. G.; Matsuoka Y.; Yu K. (2005) Molecular Basis of Replication of Duck H5N1 Influenza Viruses in a Mammalian Mouse Model. J. Virol. 79, 12058–12064. 10.1128/JVI.79.18.12058-12064.2005. 16140781 PMC1212590

[ref64] Gabriel G.; Dauber B.; Wolff T.; Planz O.; Klenk H. D.; Stech J. (2005) The Viral Polymerase Mediates Adaptation of an Avian Influenza Virus to a Mammalian Host. Proc. Natl. Acad. Sci. U. S. A. 102, 18590–18595. 10.1073/pnas.0507415102. 16339318 PMC1317936

[ref65] Taubenberger J. K.; Reid A. H.; Lourens R. M.; Wang R.; Jin G.; Fanning T. G. (2005) Characterization of the 1918 Influenza Virus Polymerase Genes. Nature 437, 889–893. 10.1038/nature04230. 16208372

[ref66] Gabriel G.; Klingel K.; Otte A.; Thiele S.; Hudjetz B.; Arman-Kalcek G.; Sauter M.; Shmidt T.; Rother F.; Baumgarte S.; Keiner B.; Hartmann E.; Bader M.; Brownlee G. G.; Fodor E.; Klenk H. D. (2011) Differential Use of Importin-α Isoforms Governs Cell Tropism and Host Adaptation of Influenza Virus. Nat. Commun. 2, 156–163. 10.1038/ncomms1158. 21245837 PMC3105303

[ref67] Gabriel G.; Herwig A.; Klenk H.-D. (2008) Interaction of Polymerase Subunit PB2 and NP with Importin A1 Is a Determinant of Host Range of Influenza A Virus. PLoS Pathog. 4, e11 10.1371/journal.ppat.0040011. 18248089 PMC2222953

[ref68] Naito T.; Momose F.; Kawaguchi A.; Nagata K. (2007) Involvement of Hsp90 in Assembly and Nuclear Import of Influenza Virus RNA Polymerase Subunits. J. Virol. 81, 1339–1349. 10.1128/JVI.01917-06. 17121807 PMC1797515

[ref69] Chase G.; Deng T.; Fodor E.; Leung B. W.; Mayer D.; Schwemmle M.; Brownlee G. (2008) Hsp90 Inhibitors Reduce Influenza Virus Replication in Cell Culture. Virology 377, 431–439. 10.1016/j.virol.2008.04.040. 18570972

[ref70] Resa-Infante P.; Paterson D.; Bonet J.; Otte A.; Oliva B.; Fodor E.; Gabriel G. (2015) Targeting Importin-A7 as a Therapeutic Approach against Pandemic Influenza Viruses. J. Virol. 89, 9010–9020. 10.1128/JVI.00583-15. 26085167 PMC4524051

[ref71] Mohl G.; Liddle N.; Nygaard J.; Dorius A.; Lyons N.; Hodek J.; Weber J.; Michaelis D. J.; Busath D. D. (2019) Novel Influenza Inhibitors Designed to Target PB1 Interactions with Host Importin RanBP5. Antiviral Res. 164, 81–90. 10.1016/j.antiviral.2019.02.003. 30742842

[ref72] He X.; Zhou J.; Bartlam M.; Zhang R.; Ma J.; Lou Z.; Li X.; Li J.; Joachimiak A.; Zeng Z.; Ge R.; Rao Z.; Liu Y. (2008) Crystal Structure of the Polymerase PAC–PB1N Complex from an Avian Influenza H5N1 Virus. Nature 454, 1123–1126. 10.1038/nature07120. 18615018

[ref73] Liu H.; Yao X. (2010) Molecular Basis of the Interaction for an Essential Subunit PA-PB1 in Influenza Virus RNA Polymerase: Insights from Molecular Dynamics Simulation and Free Energy Calculation. Mol. Pharmaceutics 7, 75–85. 10.1021/mp900131p. 19883112

[ref74] Obayashi E.; Yoshida H.; Kawai F.; Shibayama N.; Kawaguchi A.; Nagata K.; Tame J. R. H.; Park S.-Y. (2008) The Structural Basis for an Essential Subunit Interaction in Influenza Virus RNA Polymerase. Nature 454, 1127–1131. 10.1038/nature07225. 18660801

[ref75] Ghanem A.; Mayer D.; Chase G.; Tegge W.; Frank R.; Kochs G.; Garcia-Sastre A.; Schwemmle M. (2007) Peptide-Mediated Interference with Influenza A Virus Polymerase. J. Virol. 81, 7801–7804. 10.1128/JVI.00724-07. 17494067 PMC1933368

[ref76] Sugiyama K.; Obayashi E.; Kawaguchi A.; Suzuki Y.; Tame J. R. H.; Nagata K.; Park S.-Y. (2009) Structural Insight into the Essential PB1-PB2 Subunit Contact of the Influenza Virus RNA Polymerase. EMBO J. 28, 1803–1811. 10.1038/emboj.2009.138. 19461581 PMC2699363

[ref77] Yuan S.; Chu H.; Ye J.; Singh K.; Ye Z.; Zhao H.; Kao R. Y. T.; Chow B. K. C.; Zhou J.; Zheng B. J. (2017) Identification of a Novel Small-Molecule Compound Targeting the Influenza A Virus Polymerase PB1-PB2 Interface. Antiviral Res. 137, 58–66. 10.1016/j.antiviral.2016.11.005. 27840201 PMC7113721

[ref78] Massari S.; Goracci L.; Desantis J.; Tabarrini O. (2016) Polymerase Acidic Protein–Basic Protein 1 (PA–PB1) Protein–Protein Interaction as a Target for Next-Generation Anti-Influenza Therapeutics. J. Med. Chem. 59, 7699–7718. 10.1021/acs.jmedchem.5b01474. 27046062

[ref79] Engelhardt O. G.; Smith M.; Fodor E. (2005) Association of the Influenza A Virus RNA-Dependent RNA Polymerase with Cellular RNA Polymerase II. J. Virol. 79, 5812–5818. 10.1128/JVI.79.9.5812-5818.2005. 15827195 PMC1082766

[ref80] Martínez-Alonso M.; Hengrung N.; Fodor E. (2016) RNA-Free and Ribonucleoprotein-Associated Influenza Virus Polymerases Directly Bind the Serine-5-Phosphorylated Carboxyl-Terminal Domain of Host RNA Polymerase II. J. Virol. 90, 6014–6021. 10.1128/JVI.00494-16. 27099314 PMC4907247

[ref81] Lukarska M.; Fournier G.; Pflug A.; Resa-Infante P.; Reich S.; Naffakh N.; Cusack S. (2017) Structural Basis of an Essential Interaction between Influenza Polymerase and Pol II CTD. Nature 541, 117–121. 10.1038/nature20594. 28002402

[ref82] Serna Martin I.; Hengrung N.; Renner M.; Sharps J.; Martínez-Alonso M.; Masiulis S.; Grimes J. M.; Fodor E. (2018) A Mechanism for the Activation of the Influenza Virus Transcriptase. Mol. Cell 70, 1101–1110. 10.1016/j.molcel.2018.05.011. 29910112 PMC6024077

[ref83] Mistry B.; Long J. S.; Schreyer J.; Staller E.; Sanchez-David R. Y.; Barclay W. S. (2020) Elucidating the Interactions between Influenza Virus Polymerase and Host Factor ANP32A. J. Virol. 94, 1353–1369. 10.1128/JVI.01353-19. PMC7000967 31694956

[ref84] Sugiyama K.; Kawaguchi A.; Okuwaki M.; Nagata K. (2015) PP32 and APRIL Are Host Cell-Derived Regulators of Influenza Virus RNA Synthesis from CRNA. eLife 4, 8939–8958. 10.7554/eLife.08939. PMC4718810 26512887

[ref85] Staller E.; Sheppard C. M.; Neasham P. J.; Mistry B.; Peacock T. P.; Goldhill D. H.; Long J. S.; Barclay W. S. (2019) ANP32 Proteins Are Essential for Influenza Virus Replication in Human Cells. J. Virol. 93, 217–230. 10.1128/JVI.00217-19. PMC6694824 31217244

[ref86] Zhang H.; Zhang Z.; Wang Y.; Wang M.; Wang X.; Zhang X.; Ji S.; Du C.; Chen H.; Wang X. (2019) Fundamental Contribution and Host Range Determination of ANP32A and ANP32B in Influenza A Virus Polymerase Activity. J. Virol. 93, 174–191. 10.1128/JVI.00174-19. PMC6580979 30996088

[ref87] Long J. S.; Giotis E. S.; Moncorgé O.; Frise R.; Mistry B.; James J.; Morisson M.; Iqbal M.; Vignal A.; Skinner M. A.; Barclay W. S. (2016) Species Difference in ANP32A Underlies Influenza A Virus Polymerase Host Restriction. Nature 529, 101–104. 10.1038/nature16474. 26738596 PMC4710677

[ref88] Subbarao E. K.; London W.; Murphy B. R. (1993) A Single Amino Acid in the PB2 Gene of Influenza A Virus Is a Determinant of Host Range. J. Virol. 67, 1761–1764. 10.1128/JVI.67.4.1761-1764.1993. 8445709 PMC240216

[ref89] Long J. S.; Mistry B.; Haslam S. M.; Barclay W. S. (2019) Host and Viral Determinants of Influenza A Virus Species Specificity. Nat. Rev. Microbiol. 17, 67–81. 10.1038/s41579-018-0115-z. 30487536

[ref90] Gabriel G.; Fodor E. (2014) Molecular Determinants of Pathogenicity in the Polymerase Complex. Curr. Top. Microbiol. Immunol. 385, 35–60. 10.1007/82_2014_386. 25033751

[ref91] Soh Y. Q. S.; Moncla L. H; Eguia R.; Bedford T.; Bloom J. D (2019) Comprehensive Mapping of Adaptation of the Avian Influenza Polymerase Protein PB2 to Humans. eLife 8, 45079–45107. 10.7554/eLife.45079. PMC6491042 31038123

[ref92] Mänz B.; Götz V.; Wunderlich K.; Eisel J.; Kirchmair J.; Stech J.; Stech O.; Chase G.; Frank R.; Schwemmle M. (2011) Disruption of the Viral Polymerase Complex Assembly as a Novel Approach to Attenuate Influenza A Virus. J. Biol. Chem. 286, 8414–8424. 10.1074/jbc.M110.205534. 21183679 PMC3048726

[ref93] Wunderlich K.; Mayer D.; Ranadheera C.; Holler A.-S.; Mänz B.; Martin A.; Chase G.; Tegge W.; Frank R.; Kessler U.; Schwemmle M. (2009) Identification of a PA-Binding Peptide with Inhibitory Activity against Influenza A and B Virus Replication. PLoS One 4, e7517 10.1371/journal.pone.0007517. 19841738 PMC2759517

[ref94] Wunderlich K.; Juozapaitis M.; Ranadheera C.; Kessler U.; Martin A.; Eisel J.; Beutling U.; Frank R.; Schwemmle M. (2011) Identification of High-Affinity PB1-Derived Peptides with Enhanced Affinity to the PA Protein of Influenza A Virus Polymerase. Antimicrob. Agents Chemother. 55, 696–702. 10.1128/AAC.01419-10. 21135188 PMC3028788

[ref95] Muratore G.; Goracci L.; Mercorelli B.; Foeglein Á.; Digard P.; Cruciani G.; Palù G.; Loregian A. (2012) Small Molecule Inhibitors of Influenza A and B Viruses That Act by Disrupting Subunit Interactions of the Viral Polymerase. Proc. Natl. Acad. Sci. U. S. A. 109, 6247–6252. 10.1073/pnas.1119817109. 22474359 PMC3341009

[ref96] Massari S.; Nannetti G.; Goracci L.; Sancineto L.; Muratore G.; Sabatini S.; Manfroni G.; Mercorelli B.; Cecchetti V.; Facchini M.; Palù G.; Cruciani G.; Loregian A.; Tabarrini O. (2013) Structural Investigation of Cycloheptathiophene-3-Carboxamide Derivatives Targeting Influenza Virus Polymerase Assembly. J. Med. Chem. 56, 10118–10131. 10.1021/jm401560v. 24313730

[ref97] Lepri S.; Nannetti G.; Muratore G.; Cruciani G.; Ruzziconi R.; Mercorelli B.; Palu G.; Loregian A.; Goracci L. (2014) Optimization of Small-Molecule Inhibitors of Influenza Virus Polymerase: From Thiophene-3-Carboxamide to Polyamido Scaffolds. J. Med. Chem. 57, 4337–4350. 10.1021/jm500300r. 24785979

[ref98] Massari S.; Nannetti G.; Desantis J.; Muratore G.; Sabatini S.; Manfroni G.; Mercorelli B.; Cecchetti V.; Palù G.; Cruciani G.; Loregian A.; Goracci L.; Tabarrini O. (2015) A Broad Anti-Influenza Hybrid Small Molecule That Potently Disrupts the Interaction of Polymerase Acidic Protein-Basic Protein 1 (PA-PB1) Subunits. J. Med. Chem. 58, 3830–3842. 10.1021/acs.jmedchem.5b00012. 25856229

[ref99] Trist I. M. L.; Nannetti G.; Tintori C.; Fallacara A. L.; Deodato D.; Mercorelli B.; Palù G.; Wijtmans M.; Gospodova T.; Edink E.; Verheij M.; De Esch I.; Viteva L.; Loregian A.; Botta M. (2016) 4,6-Diphenylpyridines as Promising Novel Anti-Influenza Agents Targeting the PA-PB1 Protein-Protein Interaction: Structure-Activity Relationships Exploration with the Aid of Molecular Modeling. J. Med. Chem. 59, 2688–2703. 10.1021/acs.jmedchem.5b01935. 26924568

[ref100] Pagano M.; Castagnolo D.; Bernardini M.; Fallacara A. L.; Laurenzana I.; Deodato D.; Kessler U.; Pilger B.; Stergiou L.; Strunze S.; Tintori C.; Botta M. (2014) The Fight against the Influenza A Virus H1N1: Synthesis, Molecular Modeling, and Biological Evaluation of Benzofurazan Derivatives as Viral RNA Polymerase Inhibitors. ChemMedChem 9, 129–150. 10.1002/cmdc.201300378. 24285596

[ref101] Fukuoka M.; Minakuchi M.; Kawaguchi A.; Nagata K.; Kamatari Y. O.; Kuwata K. (2012) Structure-Based Discovery of Anti-Influenza Virus A Compounds among Medicines. Biochim. Biophys. Acta, Gen. Subj. 1820, 90–95. 10.1016/j.bbagen.2011.11.003. 22108550

[ref102] Muratore G.; Mercorelli B.; Goracci L.; Cruciani G.; Digard P.; Palù G.; Loregian A. (2012) Human Cytomegalovirus Inhibitor AL18 Also Possesses Activity against Influenza A and B Viruses. Antimicrob. Agents Chemother. 56, 6009–6013. 10.1128/AAC.01219-12. 22908168 PMC3486565

[ref103] Yuan S.; Chu H.; Zhao H.; Zhang K.; Singh K.; Chow B. K. C.; Kao R. Y. T.; Zhou J.; Zheng B.-J. (2016) Identification of a Small-Molecule Inhibitor of Influenza Virus via Disrupting the Subunits Interaction of the Viral Polymerase. Antiviral Res. 125, 34–42. 10.1016/j.antiviral.2015.11.005. 26593979

[ref104] Watanabe K.; Ishikawa T.; Otaki H.; Mizuta S.; Hamada T.; Nakagaki T.; Ishibashi D.; Urata S.; Yasuda J.; Tanaka Y.; Nishida N. (2017) Structure-Based Drug Discovery for Combating Influenza Virus by Targeting the PA–PB1 Interaction. Sci. Rep. 7, 9500–9512. 10.1038/s41598-017-10021-w. 28842649 PMC5573363

[ref105] Lo C.-Y.; Li O. T.-W.; Tang W.-P.; Hu C.; Wang G. X.; Ngo J. C.-K.; Wan D. C.-C.; Poon L. L.-M.; Shaw P.-C. (2018) Identification of Influenza Polymerase Inhibitors Targeting C-Terminal Domain of PA through Surface Plasmon Resonance Screening. Sci. Rep. 8, 2280–2293. 10.1038/s41598-018-20772-9. 29396435 PMC5797126

[ref106] D’Agostino I.; Giacchello I.; Nannetti G.; Fallacara A. L.; Deodato D.; Musumeci F.; Grossi G.; Palù G.; Cau Y.; Trist I. M.; Loregian A.; Schenone S.; Botta M. (2018) Synthesis and Biological Evaluation of a Library of Hybrid Derivatives as Inhibitors of Influenza Virus PA-PB1 Interaction. Eur. J. Med. Chem. 157, 743–758. 10.1016/j.ejmech.2018.08.032. 30142611

[ref107] Tintori C.; Laurenzana I.; Fallacara A. L.; Kessler U.; Pilger B.; Stergiou L.; Botta M. (2014) High-Throughput Docking for the Identification of New Influenza A Virus Polymerase Inhibitors Targeting the PA-PB1 Protein-Protein Interaction. Bioorg. Med. Chem. Lett. 24, 280–282. 10.1016/j.bmcl.2013.11.019. 24314669

[ref108] Zhang J.; Hu Y.; Foley C.; Wang Y.; Musharrafieh R.; Xu S.; Zhang Y.; Ma C.; Hulme C.; Wang J. (2018) Exploring Ugi-Azide Four-Component Reaction Products for Broad-Spectrum Influenza Antivirals with a High Genetic Barrier to Drug Resistance. Sci. Rep. 8, 4653–4667. 10.1038/s41598-018-22875-9. 29545578 PMC5854701

[ref109] Zhang J.; Hu Y.; Wu N.; Wang J. (2020) Discovery of Influenza Polymerase PA-PB1 Interaction Inhibitors Using an in Vitro Split-Luciferase Complementation-Based Assay. ACS Chem. Biol. 15, 74–82. 10.1021/acschembio.9b00552. 31714745 PMC7028398

[ref110] Massari S.; Desantis J.; Nannetti G.; Sabatini S.; Tortorella S.; Goracci L.; Cecchetti V.; Loregian A.; Tabarrini O. (2017) Efficient and Regioselective One-Step Synthesis of 7-Aryl-5-Methyl- and 5-Aryl-7-Methyl-2-Amino-[1,2,4]Triazolo[1,5-a]Pyrimidine Derivatives. Org. Biomol. Chem. 15, 7944–7955. 10.1039/C7OB02085F. 28902220

[ref111] Desantis J.; Nannetti G.; Massari S.; Barreca M. L.; Manfroni G.; Cecchetti V.; Palù G.; Goracci L.; Loregian A.; Tabarrini O. (2017) Exploring the Cycloheptathiophene-3-Carboxamide Scaffold to Disrupt the Interactions of the Influenza Polymerase Subunits and Obtain Potent Anti-Influenza Activity. Eur. J. Med. Chem. 138, 128–139. 10.1016/j.ejmech.2017.06.015. 28666191

[ref112] Nannetti G.; Massari S.; Mercorelli B.; Bertagnin C.; Desantis J.; Palù G.; Tabarrini O.; Loregian A. (2019) Potent and Broad-Spectrum Cycloheptathiophene-3-Carboxamide Compounds That Target the PA-PB1 Interaction of Influenza Virus RNA Polymerase and Possess a High Barrier to Drug Resistance. Antiviral Res. 165, 55–64. 10.1016/j.antiviral.2019.03.003. 30885750

[ref113] Reuther P.; Mänz B.; Brunotte L.; Schwemmle M.; Wunderlich K. (2011) Targeting of the Influenza A Virus Polymerase PB1-PB2 Interface Indicates Strain-Specific Assembly Differences. J. Virol. 85, 13298–13309. 10.1128/JVI.00868-11. 21957294 PMC3233147

[ref114] World Health Organization (accessed 2020-07-16) Ten threats to global health in 2019, https://www.who.int/news-room/spotlight/ten-threats-to-global-health-in-2019.

[ref115] Furuta Y.; Takahashi K.; Fukuda Y.; Kuno M.; Kamiyama T.; Kozaki K.; Nomura N.; Egawa H.; Minami S.; Watanabe Y.; Narita H.; Shiraki K. (2002) In Vitro and in Vivo Activities of Anti-Influenza Virus Compound T-705. Antimicrob. Agents Chemother. 46, 977–981. 10.1128/AAC.46.4.977-981.2002. 11897578 PMC127093

[ref116] Yuan S.; Wen L.; Zhou J. (2018) Inhibitors of Influenza A Virus Polymerase. ACS Infect. Dis. 4, 218–223. 10.1021/acsinfecdis.7b00265. 29355011

