# Supplementary material for: Inhibition of Influenza Virus Polymerase by Interfering with Its Protein–Protein Interactions
Source: ACS Infect Dis. 2020 Oct 12;7(6):1332–50. doi: 10.1021/acsinfecdis.0c00552 (PMC8204303; doi:10.1021/acsinfecdis.0c00552)
Supplement: Supplementary file 1 — id0c00552_si_001.pdf [file id0c00552_si_001.pdf]

Inhibition of Influenza Virus Polymerase by Interfering with Its Protein-Protein Interactions

Serena Massari,[a*] Jenny Desantis,[b] Maria Giulia Nizi,[a] Violetta Cecchetti,[a] Oriana Tabarrini.[a*]

[a] *Department of Pharmaceutical Sciences, University of Perugia, 06123 Perugia Italy;*
[b] *Department of Chemistry, Biology and Biotechnology, University of Perugia, 06123, Perugia Italy.*

* *Address correspondence to these authors at the Department of Pharmaceutical Science, University of Perugia, Via del Liceo 1, 06123 Perugia, Italy; For SM: Tel: +390755855134; E-mail: serena.massari@unipg.it. For OT: Tel: +390755855139; E-mail, oriana.tabarrini@unipg.it.*

Table of Contents

Figure S1. Binding pose of compounds **1-5** and **7-10** S2
Figure S2. Binding pose of compounds **6** S2
Figure S3. Binding pose of compound **14** S3
Figure S4. Binding pose of compound **15** S3
Figure S5. Binding pose of compound **19** S4
Figure S6. Binding pose of compound **21** S5
Figure S7. Binding pose of compound **23** S5
Figure S8. Binding pose of an analogue of compound **32** S6

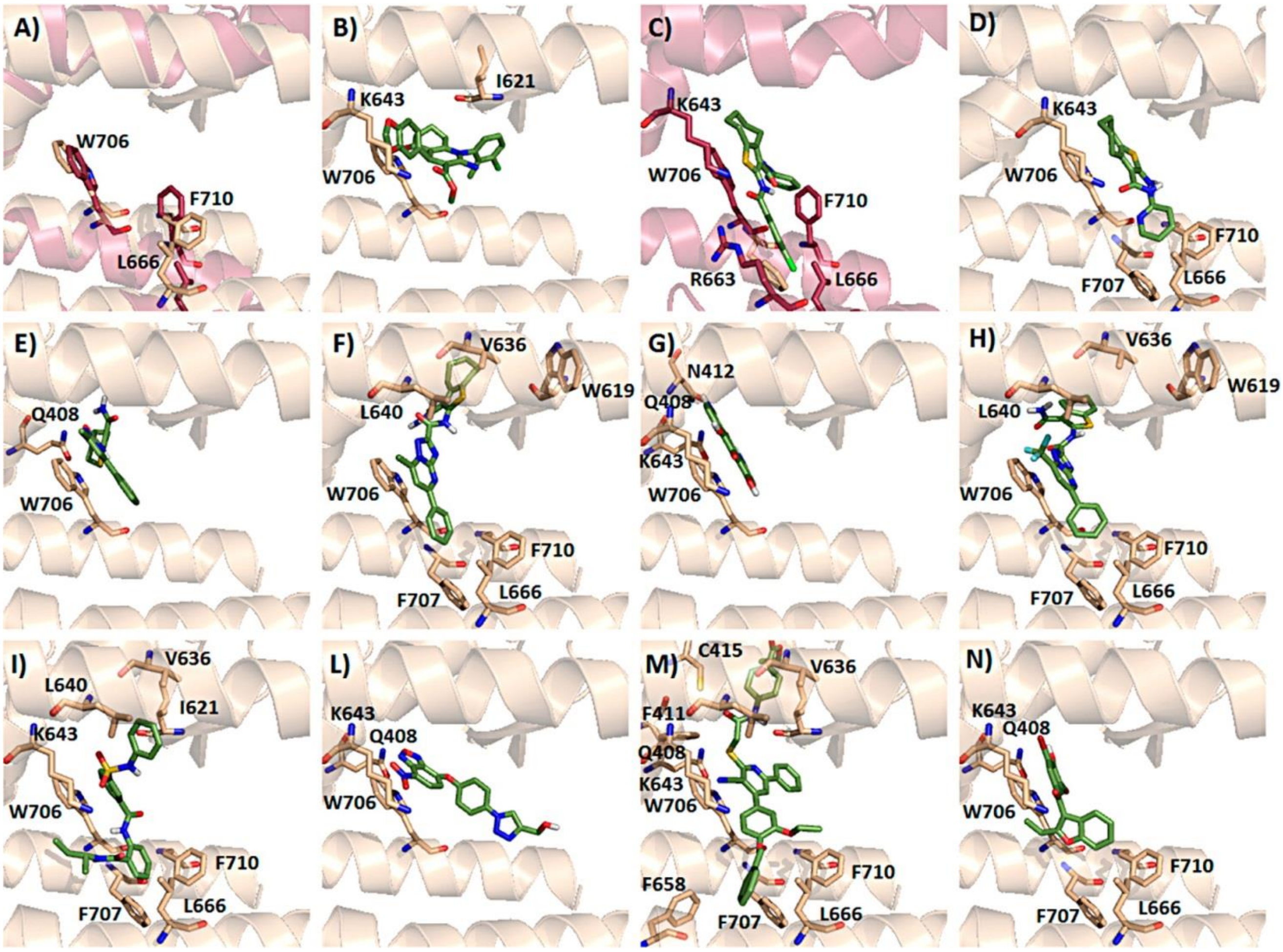

Figure S1. Key residues located in the PA cavity (A, pdb: 3CM8) and FLAP binding pose for compounds **1** (B), **2** (C), **3** (H), **4** (E), **5** (F), **7** (I), **8** (L), **9** (N), and **10** (G). (D) and (M) show the binding pose for two compounds not mentioned in this review. Reproduced from [Massari, S. Goracci, L.; Desantis, J.; Tabarrini, O. Polymerase Acidic Protein-Basic Protein 1 (PA-PB1) Protein-protein interaction as a target for next-generation anti-influenza therapeutics. *J. Med. Chem.* **2016**, *59*, 7699-7718].[1] Copyright [2016] American Chemical Society.

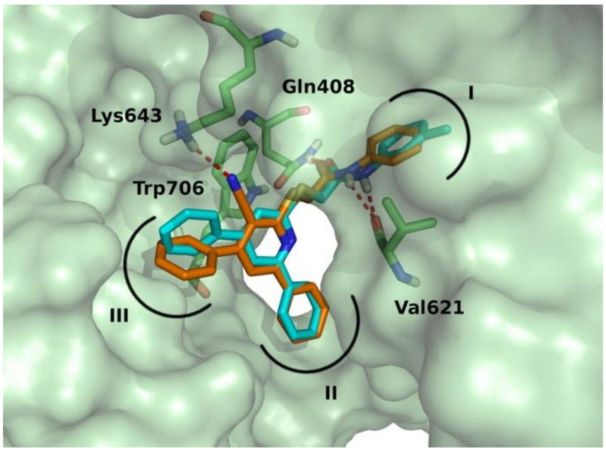

Figure S2. Docking pose for compound **6** (cyan sticks) within the PAc (pdb: from docking simulations. Reproduced from [Trist, I. M.; Nannetti, G.; Tintori, C.; Fallacara, A. L.; Deodato, D.; Mercorelli, B.; Palù, G.; Wijtmans, M.; Gospodova, T.; Edink, E.; Verheij, M.; de Esch, I.; Viteva, L.; Loregian, A.; Botta, M. 4,6-Diphenylpyridines as promising novel anti-influenza agents targeting the PA-PB1 protein-protein interaction: structure-activity relationships exploration with the aid of molecular modeling. *J. Med. Chem.* **2016**, *59*, 2688-26703].[2] Copyright [2016] American Chemical Society.

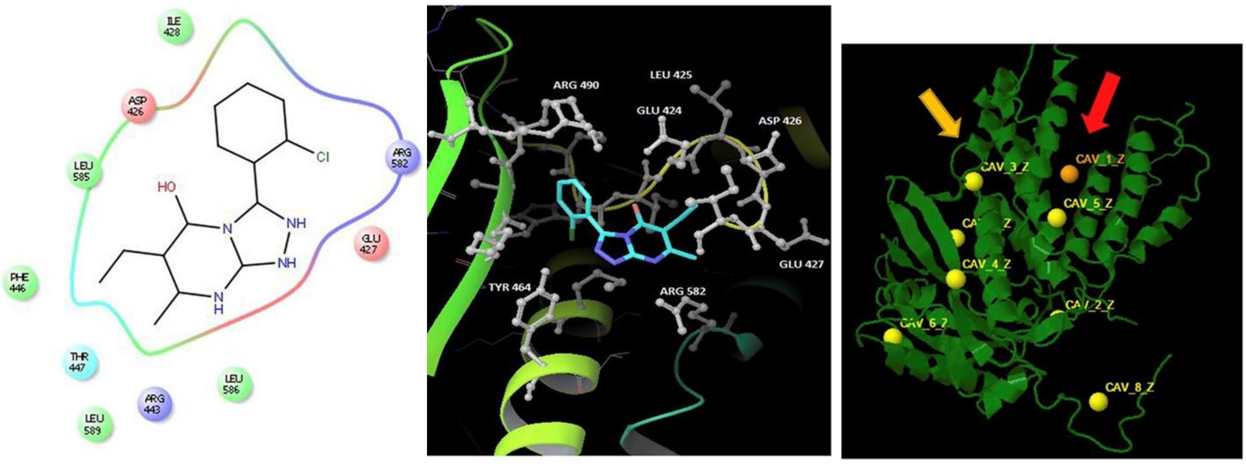

Figure S3. Docking pose for compound **14** (left and center) and binding site of compound **14** (yellow arrow) and PB1 binding site (red arrow) (right) within the PA_C (pdb: 3CM8). Reproduced with permission from [Yuan, S.; Chu, H.; Zhao, H.; Zhang, K.; Singh, K.; Chow, B. K. C.; Kao, R. Y. T.; Zhou, J.; Zheng, B.-J. Identification of a small-molecule inhibitor of influenza virus via disrupting the subunits interaction of the viral polymerase. *Antiviral Res.* **2016**, *125*, 34–42.][3] Copyright [2015] Elsevier B.V.

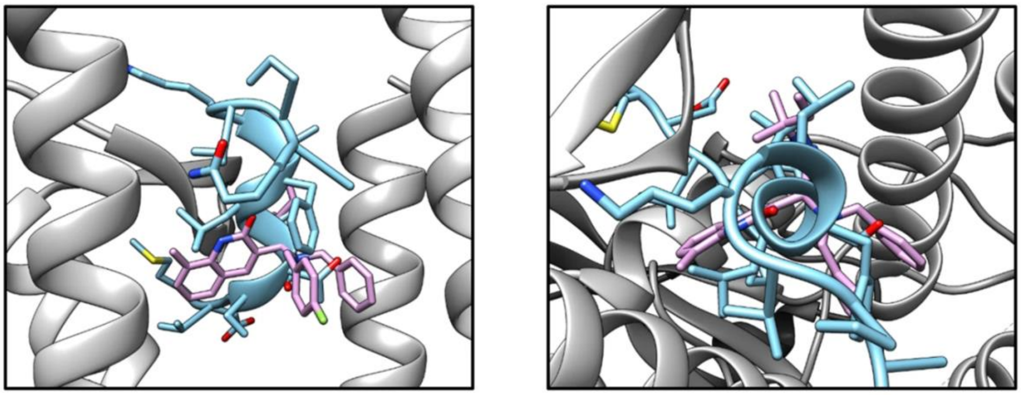

Figure S4. Docking pose for compound **15** within the PA_C based on in silico calculations, side view (left) and top view (right); crystal structure of PA (pdb: 2ZNL, grey) in complex with PB1 (light blue) and binding structure of compound **15** (pink) from docking simulation were overlaid. Reproduced with permission from [Watanabe, K.; Ishikawa, T.; Otaki, H.; Mizuta, S.; Hamada, T.; Nakagaki, T.; Ishibashi, D.; Urata, S.; Yasuda, J.; Tanaka, Y.; Nishida, N. Structure-based drug discovery for combating influenza virus by targeting the PA–PB1 interaction. *Sci. Rep.* **2017**, *7* (1), 9500.][4] Copyright [2017] Springer Nature.

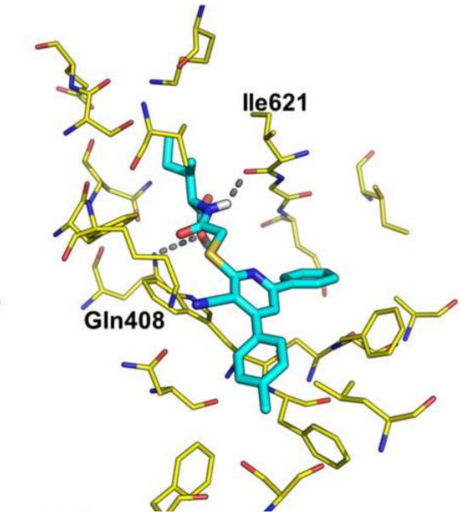

Figure S5. Docking pose for compound **19** (cyan stick). The crystallographic structure of PA in complex with PB1 (pdb: 4WSB) is shown as yellow lines. Polar interactions are highlighted by grey dashed lines. Only residues within 4 Å from the ligands are showed; non-polar H atoms are omitted. Reproduced with permission from [D'Agostino, I.; Giacchello, I.; Nannetti, G.; Fallacara, A. L.; Deodato, D.; Musumeci, F.; Grossi, G.; Palù, G.; Cau, Y.; Trist, I. M.; Loregian, A.; Schenone, S.; Botta, M. Synthesis and biological evaluation of a library of hybrid derivatives as inhibitors of influenza virus PA-PB1 interaction. *Eur. J. Med. Chem.* **2018**, *157*, 743-758].[5] Copyright [2018] Elsevier Masson SAS.

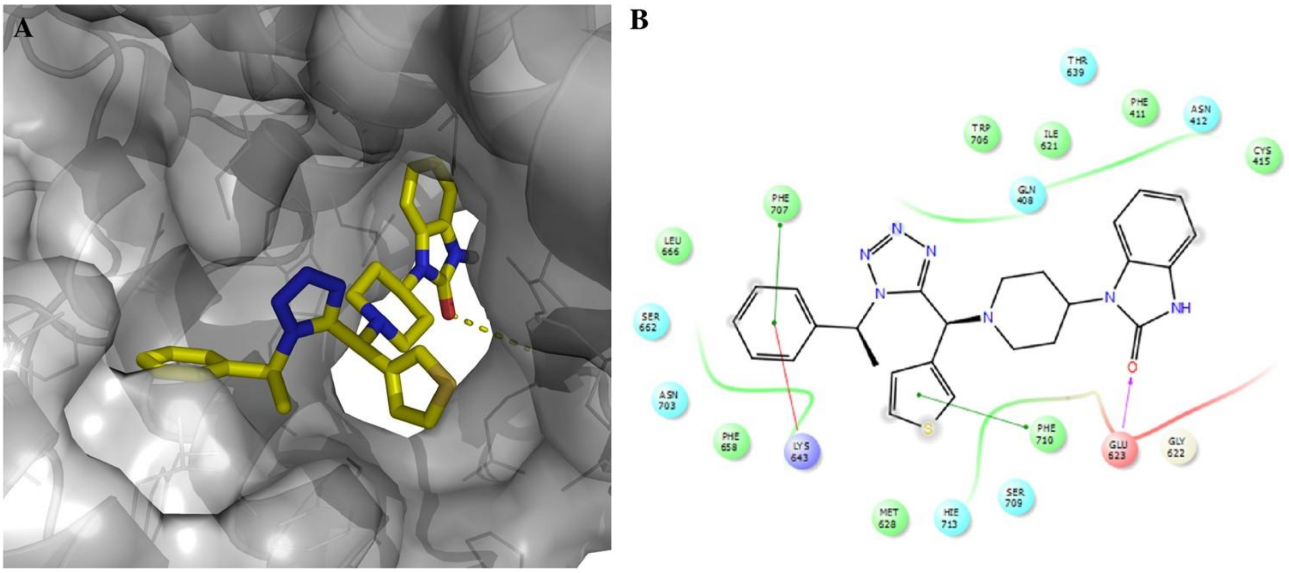

Figure S6. Surface view of docking pose for compound **21** in the PB1$_N$-binding pocket in PA$_C$ (A) and its ligand interaction diagram with residues in the binding site (B). Reproduced with permission from [Zhang, J.; Hu, Y.; Foley, C.; Wang, Y.; Musharrafieh, R.; Xu, S.; Zhang, Y.; Ma, C.; Hulme, C.; Wang, J. Exploring Ugi-azide four-component reaction products for broad-spectrum influenza antivirals with a high genetic barrier to drug resistance. *Sci. Rep.* **2018**, *8* (1), 4653].[6] Copyright [2018] Springer Nature.

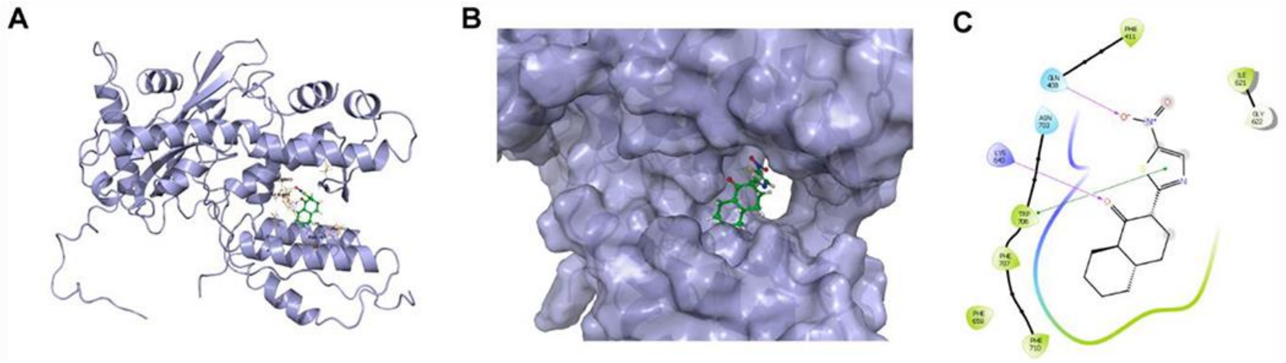

Figure S7. Best docking pose of compound **23** in PA$_C$ cavity from PA–PB1 complex (pdb: 3CM8) (A), its surface view (B) and its ligand interaction diagram (C). Reproduced from [Zhang, J.; Hu, Y.; Wu, N.; Wang, J. Discovery of influenza polymerase PA-PB1 interaction inhibitors using an in vitro split-luciferase complementation-based assay. *ACS Chem. Biol.* **2020**, *15* (1), 74–82].[7] Copyright [2020] American Chemical Society.

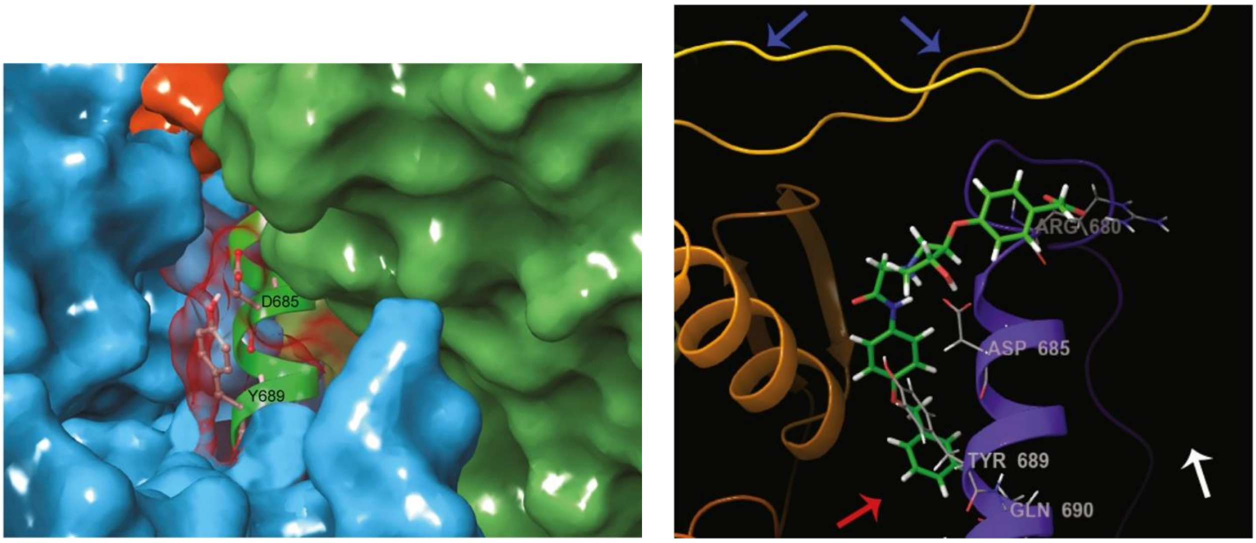

Figure S8. Proposed binding site of compound **5** (original number reported in the manuscript by Mohl et al., structure is reported below), a strict analogue of compound **32** (left): PA-PB1 complex (pdb: 4WSB, blue surface) in complex with RanBP5 homologue Kap121p (pdb: 3W3Z, green surface) and key residues D685 and Y689 of PB1 anchor helix (transparent red surface); view of the binding site of compound **5** docked (right): the bipartite NLS (blue arrows), RanBP5 binding interface (white arrow), and PB2 binding site (red arrow). Reproduced with permission from [Mohl, G., Liddle, N., Nygaard, J., Dorius, A., Lyons, N., Hodek, J., Weber, J., Michaelis, D. J., and Busath, D. D. (2019) Novel influenza inhibitors designed to target PB1 interactions with host importin RanBP5. *Antiviral Res.* *164*, 81–90].[8] Copyright [2019] Elsevier B.V.

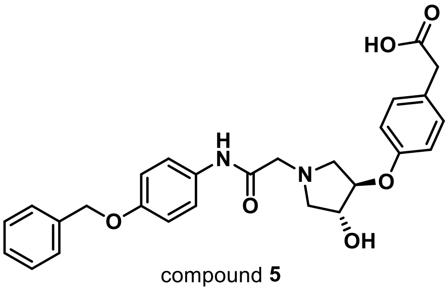

compound **5**

References

(1) Massari, S.; Nannetti, G.; Desantis, J.; Muratore, G.; Sabatini, S.; Manfroni, G.; Mercorelli, B.; Cecchetti, V.; Palù, G.; Cruciani, G.; Loregian, A.; Goracci, L.; Tabarrini, O. A Broad Anti-Influenza Hybrid Small Molecule That Potently Disrupts the Interaction of Polymerase Acidic Protein-Basic Protein 1 (PA-PB1) Subunits. *J. Med. Chem.* **2015**, *58*, 3830–3842. https://doi.org/10.1021/acs.jmedchem.5b00012.

(2) Trist, I. M. L.; Nannetti, G.; Tintori, C.; Fallacara, A. L.; Deodato, D.; Mercorelli, B.; Palù, G.; Wijtmans, M.; Gospodova, T.; Edink, E.; Verheij, M.; De Esch, I.; Viteva, L.; Loregian, A.; Botta, M. 4,6-Diphenylpyridines as Promising Novel Anti-Influenza Agents Targeting the PA-PB1 Protein-Protein Interaction: Structure-Activity Relationships Exploration with the Aid of Molecular Modeling. *J. Med. Chem.* **2016**, *59*, 2688–2703. https://doi.org/10.1021/acs.jmedchem.5b01935.

(3) Yuan, S.; Chu, H.; Zhao, H.; Zhang, K.; Singh, K.; Chow, B. K. C.; Kao, R. Y. T.; Zhou, J.; Zheng, B.-J. Identification of a Small-Molecule Inhibitor of Influenza Virus via Disrupting the Subunits Interaction of the Viral Polymerase. *Antiviral Res.* **2016**, *125*, 34–42. https://doi.org/10.1016/j.antiviral.2015.11.005.

(4) Watanabe, K.; Ishikawa, T.; Otaki, H.; Mizuta, S.; Hamada, T.; Nakagaki, T.; Ishibashi, D.; Urata, S.; Yasuda, J.; Tanaka, Y.; Nishida, N. Structure-Based Drug Discovery for Combating Influenza Virus by Targeting the PA–PB1 Interaction. *Sci. Rep.* **2017**, *7*, 9500–9512. https://doi.org/10.1038/s41598-017-10021-w.

(5) D’Agostino, I.; Giacchello, I.; Nannetti, G.; Fallacara, A. L.; Deodato, D.; Musumeci, F.; Grossi, G.; Palù, G.; Cau, Y.; Trist, I. M.; Loregian, A.; Schenone, S.; Botta, M. Synthesis and Biological Evaluation of a Library of Hybrid Derivatives as Inhibitors of Influenza Virus PA-PB1 Interaction. *Eur. J. Med. Chem.* **2018**, *157*, 743–758. https://doi.org/10.1016/j.ejmech.2018.08.032.

(6) Zhang, J.; Hu, Y.; Foley, C.; Wang, Y.; Musharrafieh, R.; Xu, S.; Zhang, Y.; Ma, C.; Hulme,

C.; Wang, J. Exploring Ugi-Azide Four-Component Reaction Products for Broad-Spectrum Influenza Antivirals with a High Genetic Barrier to Drug Resistance. *Sci. Rep.* **2018**, *8*, 4653–4667. https://doi.org/10.1038/s41598-018-22875-9.

(7) Zhang, J.; Hu, Y.; Wu, N.; Wang, J. Discovery of Influenza Polymerase PA-PB1 Interaction Inhibitors Using an in Vitro Split-Luciferase Complementation-Based Assay. *ACS Chem. Biol.* **2020**, *15*, 74–82. https://doi.org/10.1021/acschembio.9b00552.

(8) Mohl, G.; Liddle, N.; Nygaard, J.; Dorius, A.; Lyons, N.; Hodek, J.; Weber, J.; Michaelis, D. J.; Busath, D. D. Novel Influenza Inhibitors Designed to Target PB1 Interactions with Host Importin RanBP5. *Antiviral Res.* **2019**, *164*, 81–90. https://doi.org/10.1016/j.antiviral.2019.02.003.
